# Integrated Engineering of CAR‐T Cells for Solid Tumours

**DOI:** 10.1111/cpr.70263

**Published:** 2026-07-14

**Authors:** Chao Yang, Tan Li, Ping He, Zhijie Zhao, Shenglong Li, Liming Wang

**Affiliations:** ^1^ Trauma Center and Department of Burns The First Hospital of China Medical University Shenyang Liaoning China; ^2^ Department of Cardiovascular Ultrasound The First Hospital of China Medical University Shenyang Liaoning China; ^3^ Department of Laboratory Medical Center General Hospital of Northern Theater Command Shenyang Liaoning China; ^4^ Department of Plastic and Reconstructive Surgery Shanghai Ninth People's Hospital, Shanghai Jiao Tong University School of Medicine Shanghai China; ^5^ Second Ward of Bone and Soft Tissue Tumor Surgery, Cancer Hospital of Dalian University of Technology, Cancer Hospital of China Medical University Liaoning Cancer Hospital & Institute Shenyang Liaoning China; ^6^ Department of Thoracic Surgery The First Hospital of China Medical University Shenyang Liaoning China

**Keywords:** CAR‐T cell, metabolic reprogramming, regional delivery, solid tumours, tumour microenvironment (TME)

## Abstract

Chimeric antigen receptor (CAR) T‐cell therapy has achieved durable efficacy in hematologic malignancies but encounters persistent obstacles in solid tumours, including antigen heterogeneity, a suppressive tumour microenvironment (TME), and intrinsic T‐cell dysfunction. This review examines the transition from single‐axis engineering to an integrated framework that addresses these hurdles in sequence. We delineate how next‐generation CAR‐T cells are designed for precise spatiotemporal activation through logic‐gated and pharmacologically regulatable receptors, while being reinforced by metabolic and epigenetic reprogramming to resist TME‐driven exhaustion. We also assess strategies that actively reshape the immunosuppressive TME, including depletion of regulatory cell populations, blockade of ‘don't eat me’ signals, and the use of biomaterial scaffolds for locoregional delivery. The synthesis of controllable activation, intrinsic resilience, and extrinsic TME modulation is defining a class of adaptive therapeutic systems. Clinical implementation of this approach requires careful management of toxicities, notably cytokine release syndrome (CRS), and support from advanced monitoring technologies. Progress will depend on rational combinations that move beyond isolated optimisations, enabling cellular therapies to dynamically respond to evolving tumour ecosystems and narrowing the efficacy gap between hematologic and solid cancers.

## Graphical abstract

Solid tumours pose multifactorial barriers including antigen heterogeneity, immunosuppressive microenvironment, and poor T‐cell trafficking, limiting CAR‐T efficacy compared to hematologic malignancies. Integrated engineering strategies are essential, combining logic‐gated receptors for precision, metabolic‐epigenetic reprogramming for resilience, and TME‐remodelling approaches to overcome suppressive barriers. Target selection must balance specificity and coverage, utilising tumour‐restricted antigens, hematologic checkpoints, and neoantigens while employing dual‐targeting systems to prevent antigen escape. Advanced technologies enable rational design, from high‐resolution imaging and spatial multi‐omics to CRISPR screening and base editing, driving the development of next‐generation, adaptable CAR‐T cell therapies.

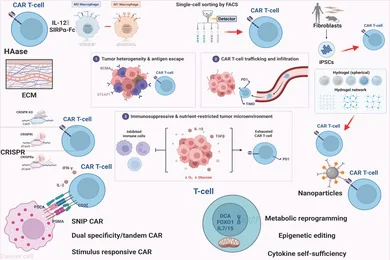

**Abbreviations**
AdCAR: adapter chimeric antigen receptor
aLNPs: antigen‐presenting cell‐mimetic lipid nanoparticles
AML: acute myeloid leukaemia
ASCT: autologous stem cell transplantation
B7‐H3: B7 homologue 3
BCA: B‐cell aplasia
BCAA: branched‐chain amino acid
BCKDK: branched‐chain ketoacid dehydrogenase kinase
BCMA: B‐cell maturation antigen
CAR: chimeric antigen receptor
CD133: cluster of differentiation 133 (Prominin‐1)
CD276: cluster of differentiation 276
CIITA: class II transactivator
CIK cells: cytokine‐induced killer cells
cilta‐cel: ciltacabtagene autoleucel
CLDN18.2: claudin 18.2
CMC‐21: CaMnCO_3_/IL‐21 nanoparticles
CNS: central nervous system
CRISPR: clustered regularly interspaced short palindromic repeats
CRS: cytokine release syndrome
DCA: dichloroacetate
DIPG: diffuse intrinsic pontine glioma
EDA: extra domain A of fibronectin
EGFRvIII: epidermal growth factor receptor variant III
ENI: electroactive nano‐injection
FAP: fibroblast activation protein
FMANAC: TME‐responsive nanoimmunomodulator
GelMA: gelatin methacrylamide
GPA33: glycoprotein A33
GPC2: glypican‐2
GPX4: glutathione peroxidase 4
HBOPM: high‐throughput Bessel light‐sheet oblique plane microscopy
HDAC: histone deacetylase
HSPCs: haematopoietic stem and progenitor cells
ide‐cel: idecabtagene vicleucel
iPSC: induced pluripotent stem cell
MDSCs: myeloid‐derived suppressor cells
MHC: major histocompatibility complex
MPM: malignant pleural mesothelioma
MSLN: mesothelin
MWA: microwave ablation
nfP2X7: nonfunctional P2X7 receptor
NK cell: natural killer cell
NMOSD: neuromyelitis optica spectrum disorder
OAds: oncolytic adenoviruses
OXPHOS: oxidative phosphorylation
P4HA1: prolyl 4‐hydroxylase subunit alpha 1
pCAR: plasmid DNA encoding CAR
PD‐1: programmed cell death protein 1
PEGDA: polyethylene glycol diacrylate
PGA: polyglutamate
PLGA: poly(lactic‐co‐glycolic acid)
PPZ: pantoprazole
PROs: patient‐reported outcomes
PS: phosphatidylserine
PSMA: prostate‐specific membrane antigen
RevCAR: reverse chimeric antigen receptor
scFvs: single‐chain variable fragments
scRNA‐seq: single‐cell RNA sequencing
SMLM: single‐molecule localisation microscopy
TAA: tumour‐associated antigen
TALEN: transcription activator‐like effector nuclease
TAMs: tumour‐associated macrophages
TCA cycle: tricarboxylic acid cycle
Tcm: central memory T cell
TENG: triboelectric nanogenerator
TIM‐3: T‐cell immunoglobulin and mucin‐domain containing‐3
TME: tumour microenvironment
TNBC: triple‐negative breast cancer
TNFRSF17: TNF receptor superfamily member 17
Tregs: regulatory T cells
TRM: tissue‐resident memory T cell
TSPs: toroidal spiral particles
UCB: umbilical cord blood
VISTA: V‐domain Ig suppressor of T cell activation
Zip18R: leucine zipper‐stabilised IL‐18 receptor

## 1 Introduction

**FIGURE 1:**
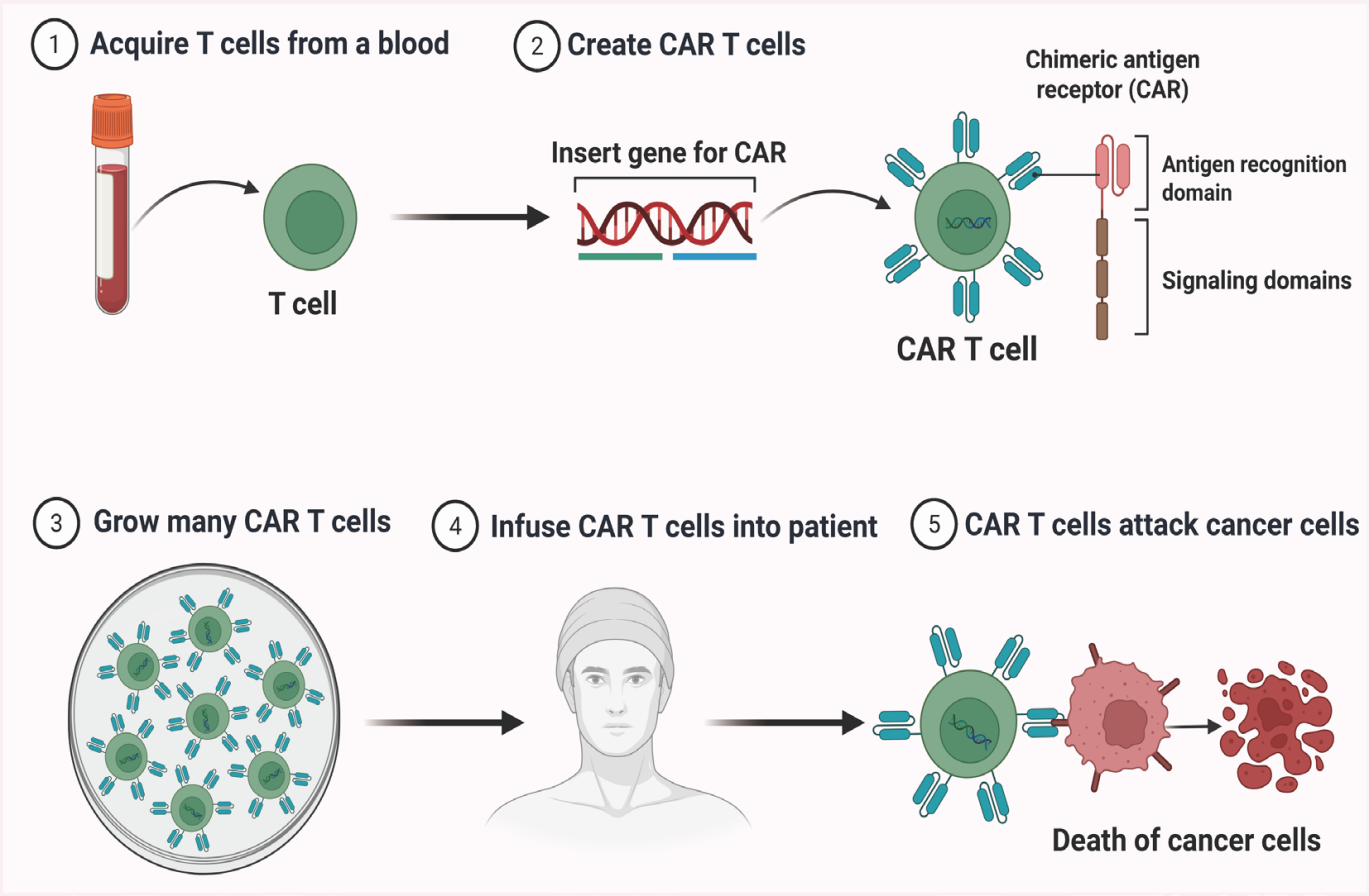
Procedural pipeline of CAR T‐cell engineering and therapeutic administration. This figure provides a high‐fidelity overview of the CAR‐T manufacturing‐to‐treatment axis, a pioneering frontier in precision oncology. The illustrated workflow is designed for multifaceted utility: It may be deployed in its integrated form to depict the end‐to‐end therapeutic cycle or adapted to elucidate specific modular components of the CAR‐T landscape.

Chimeric antigen receptor (CAR) T‐cell therapy has reshaped cancer treatment, achieving deep and durable remissions—and in some cases, functional cures—in relapsed/refractory B‐cell malignancies such as acute lymphoblastic leukaemia and lymphomas, highlighting the remarkable therapeutic potential of engineered T cells [1, 2]. To date, more than 10 CAR‐T‐cell products targeting CD19 or B‐cell maturation antigen (BCMA) have received regulatory approval worldwide, solidifying their role in the treatment of hematologic malignancies [3, 4, 5, 6, 7]. Extending these successes to solid tumours, however, has proven considerably more difficult. Although recent trials have reported activity in selected indications—including neuroblastoma, gliomas, and gastrointestinal cancers—overall efficacy in most solid malignancies remains substantially lower than that observed in hematologic indications [8, 9, 10, 11, 12, 13, 14, 15]. This efficacy gap reflects a distinct set of interdependent biological constraints that are characteristic of solid tumours. These clinical successes are underpinned by a tightly coordinated manufacturing‐to‐infusion pipeline spanning leukapheresis, viral or non‐viral CAR gene transfer, ex vivo expansion with quality‐control testing, and patient reinfusion (Figure 1).

**FIGURE 2:**
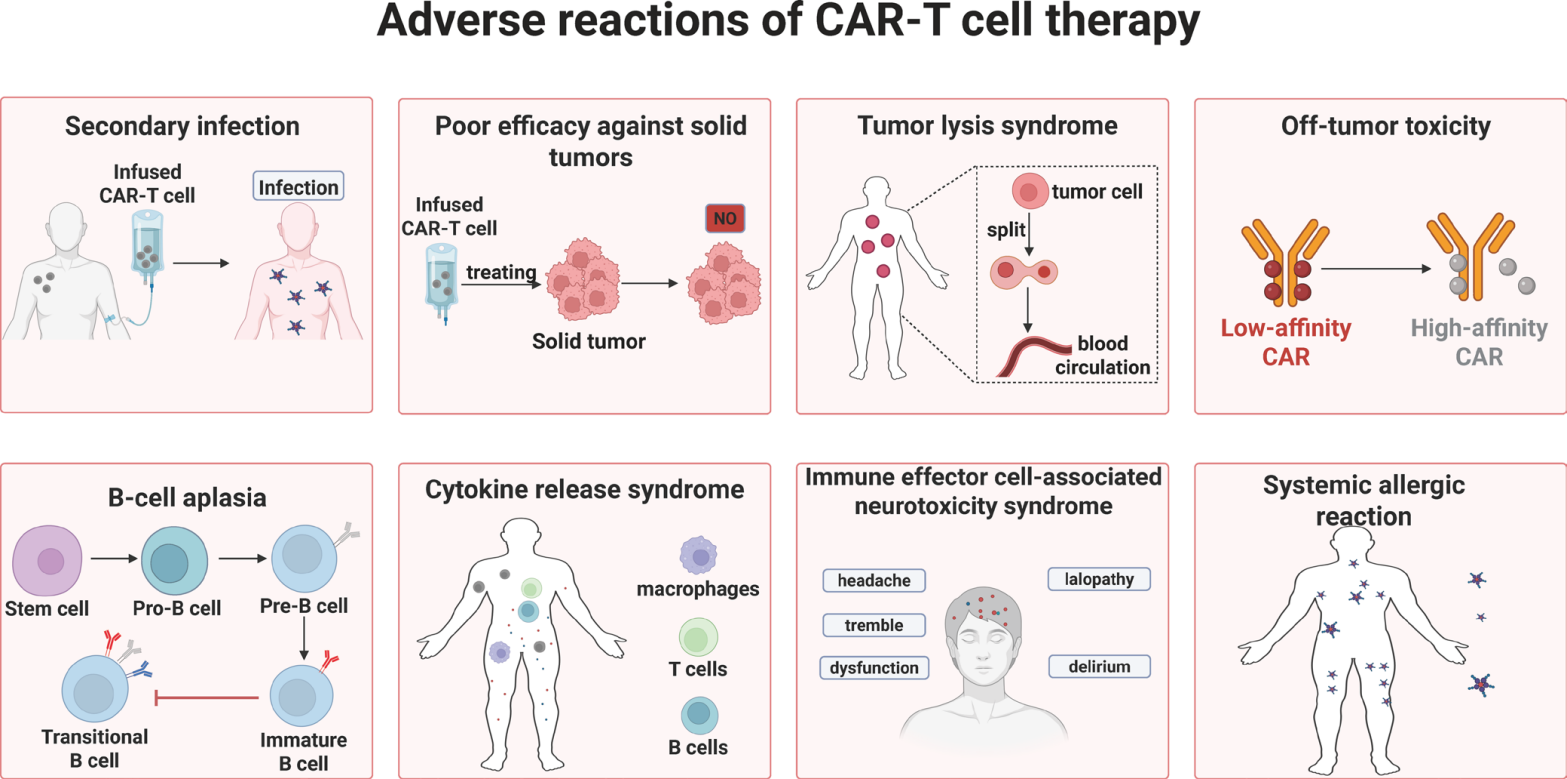
Major challenges and adverse reactions associated with CAR‐T cell therapy. CAR‐T cell therapy is associated with several clinical challenges and adverse events, including poor efficacy against solid tumours and the risk of secondary infections following infusion. Off‐tumour toxicity may arise from the systemic circulation of tumour‐derived factors, while the balance between low‐affinity and high‐affinity chimeric antigen receptor (CAR) designs remains critical for targeting precision. Common systemic toxicities include B‐cell aplasia due to the depletion of B‐cell lineages, tumour lysis syndrome (TLS), and cytokine release syndrome (CRS) mediated by the interaction of macrophages, T cells, and B cells. Furthermore, patients may experience immune effector cell‐associated neurotoxicity syndrome (ICANS)—characterized by symptoms such as headache, tremors, delirium, lalopathy, and cognitive dysfunction—as well as systemic allergic reactions.

Solid tumours impose a series of multifactorial barriers to CAR‐T‐cell activity. Central among these is target antigen selectivity: truly tumour‐restricted antigens—those with high intratumoural expression and negligible presence in essential normal tissues—are uncommon [16, 17]. Furthermore, antigen heterogeneity and therapy‐induced antigen loss or downregulation—documented for targets such as BCMA and STEAP1—render single‐antigen CAR‐T‐cell strategies susceptible to immune escape [18, 19, 20, 21, 22]. The tumour microenvironment (TME) constitutes a multifaceted immunosuppressive barrier. A dense extracellular matrix and abnormal vasculature impede T‐cell trafficking and infiltration, while hypoxia, acidosis, and nutrient scarcity create profoundly adverse biochemical conditions [23, 24, 25]. In addition, abundant immunosuppressive populations—including regulatory T cells (Tregs) and tumour‐associated macrophages (TAMs)—curtail CAR‐T‐cell proliferation, cytotoxic function, and persistence through both contact‐dependent mechanisms and inhibitory cytokine secretion. This progressively induces a state of functional exhaustion in tumour‐infiltrating T cells, characterised by sustained expression of inhibitory receptors, loss of effector capacity, and dysregulated metabolic programming [26, 27, 28, 29, 30, 31, 32, 33]. Collectively, these biological constraints manifest clinically as a spectrum of challenges and toxicities that narrow the therapeutic window of CAR‐T‐cell therapy in solid tumours (Figure 2).

**FIGURE 3:**
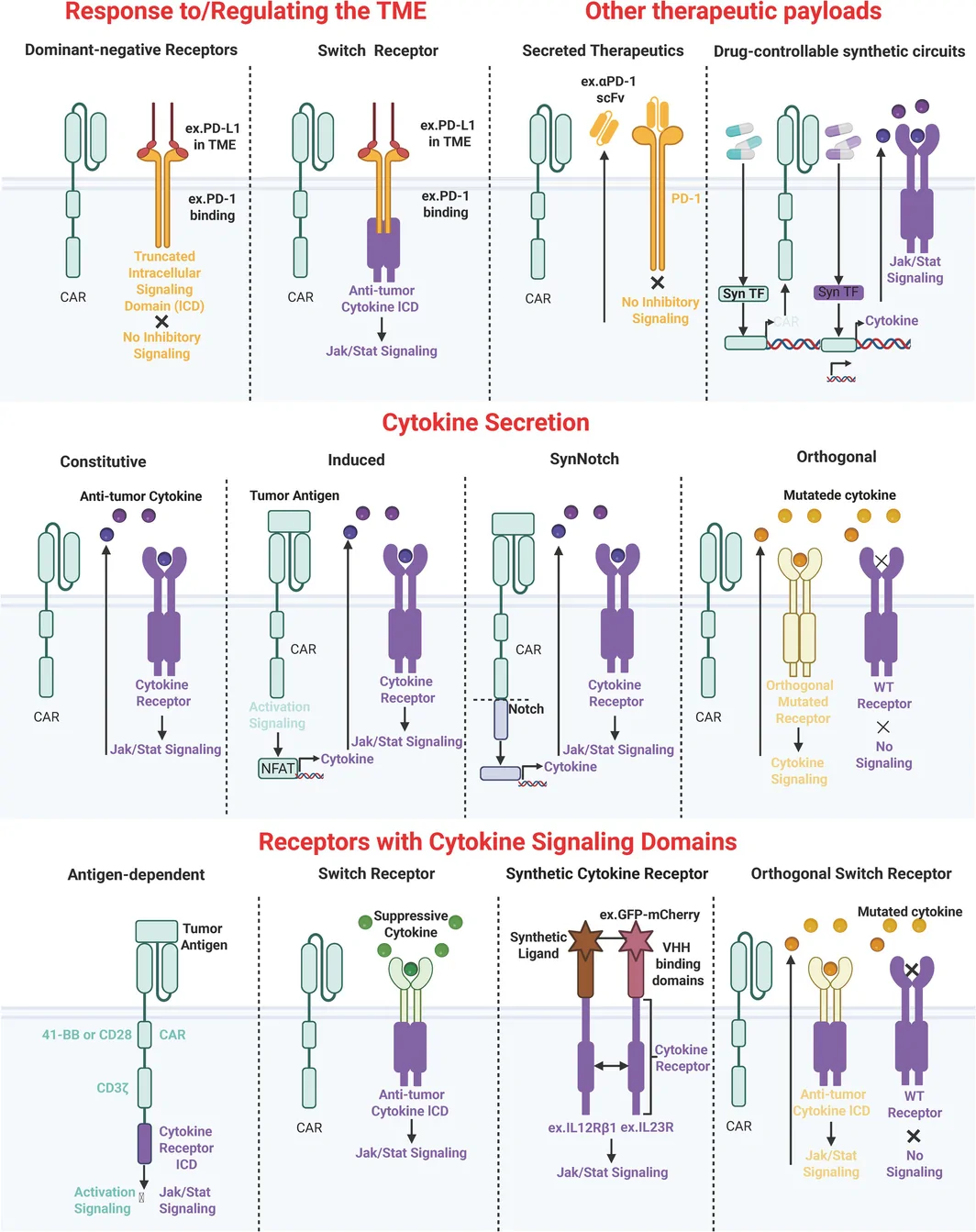
Synthetic biology strategies for modulating the tumour microenvironment (TME) and enhancing CAR T‐cell function. Various synthetic biology strategies for responding to and regulating the TME are illustrated. These include dominant‐negative receptors that use truncated intracellular domains to block inhibitory signalling, and switch receptors designed to rewire suppressive inputs into stimulatory Jak/Stat signalling. The diagram also depicts secreted therapeutics and drug‐controllable circuits utilizing synthetic transcription factors (Syn TF) for temporal control of therapeutic payloads. Furthermore, precise cytokine regulation is achieved through constitutive, antigen‐induced (NFAT‐mediated), or synthetic Notch (synNotch)‐gated expression systems, as well as orthogonal systems employing mutated cytokine‐receptor pairs to ensure signalling specificity without cross‐reactivity to wild‐type receptors.

In response to these formidable barriers, the field has pursued a diverse array of engineering strategies, achieving incremental but meaningful gains. On the targeting axis, bispecific CARs, tandem CARs, and logic‐gated circuits are designed to enhance recognition specificity and mitigate antigen heterogeneity [34, 35, 36, 37]. To strengthen cellular performance, approaches such as cytokine co‐expression, disruption of inhibitory receptors, and incorporation of pro‐survival signals have improved persistence and reduced exhaustion, albeit to varying extents [38, 39, 40]. To address the TME, CAR‐T cells engineered to express matrix‐degrading enzymes or secrete immunomodulatory factors have shown preclinical activity [41, 42]. Nevertheless, most of these interventions emphasise a single dimension and often entail significant trade‐offs. For instance, amplifying CAR signalling can increase the risk of cytokine release syndrome (CRS); promoting sustained expansion may accelerate terminal differentiation and exhaustion; and achieving high local concentrations of immunomodulators risks triggering off‐tumour inflammation [43, 44, 45]. These observations collectively indicate that isolated optimisation of individual components is unlikely to overcome the coordinated, multi‐layered defences of solid tumours. To address these barriers, investigators have applied synthetic biology to reprogram CAR T cells (Figure 3). Approaches such as dominant‐negative and switch receptors, drug‐tunable circuits, and antigen‐ or synNotch‐gated cytokine programs expand functional control, but many remain single‐axis interventions that incur potency–toxicity trade‐offs.

**FIGURE 4:**
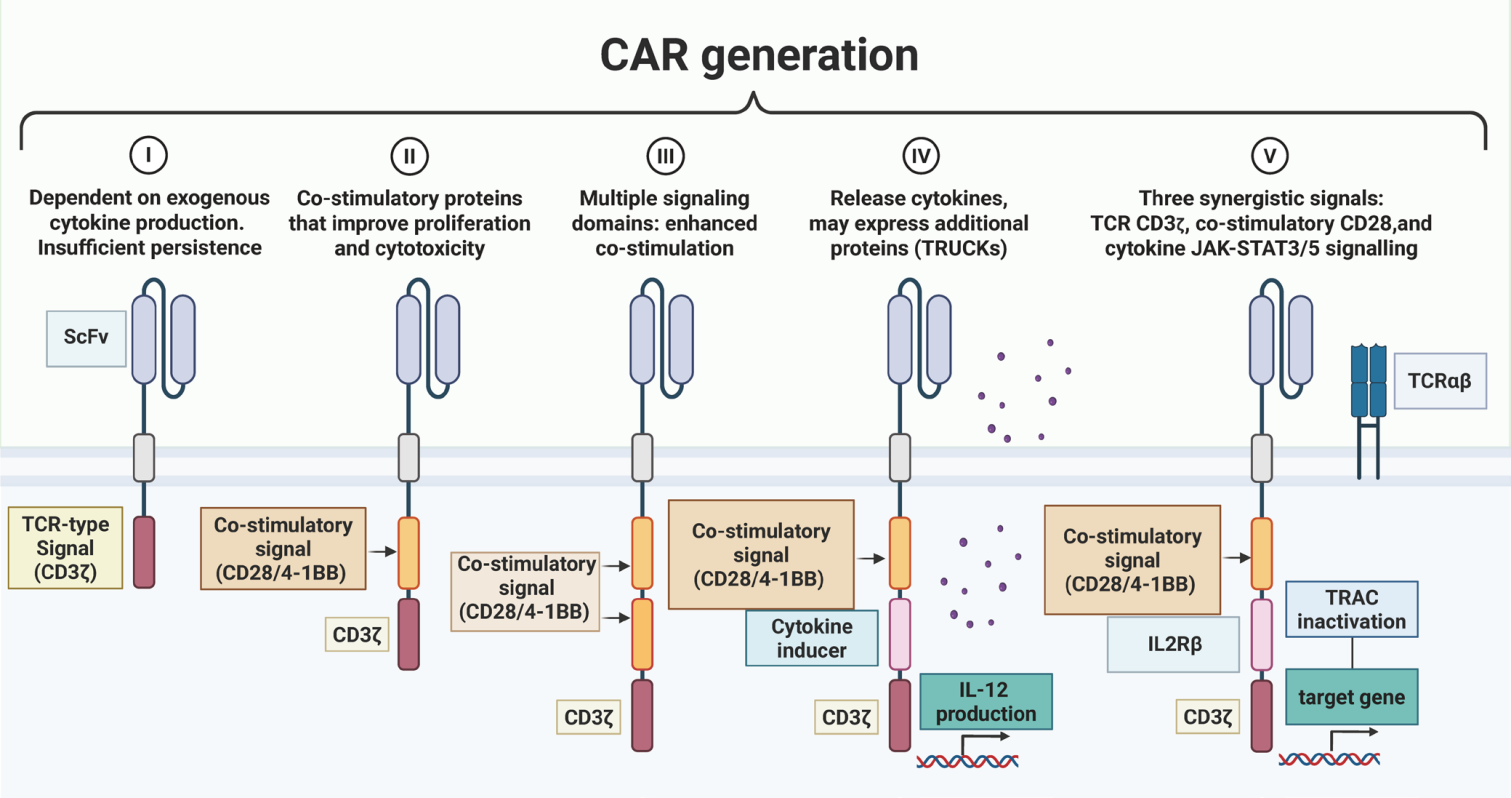
Iterative generations and structural evolution of chimeric antigen receptors (CARs). The structural development of CAR‐T cells is categorized into five generations designed to optimize therapeutic persistence and cytotoxic efficacy. Generation I features an single‐chain variable fragment (scFv) linked to a CD3ζ signalling domain but is limited by a dependence on exogenous cytokines and insufficient persistence. Generations II and III incorporate one or more co‐stimulatory domains, such as CD28 or 4‐1BB, to improve T‐cell proliferation and anti‐tumour activity. Generation IV CARs, also known as TRUCKs, are engineered to release cytokines (e.g., IL‐12) through the inclusion of cytokine inducers for localized immune modulation. Finally, Generation V integrates three synergistic signals—CD3ζ, co‐stimulatory CD28, and JAK‐STAT3/5 signalling via IL2Rβ domains—while often utilizing TRAC inactivation and TCRαβ components to further refine the therapeutic output.

Effective therapy will therefore require an integrated redesign, shifting from single‐target ‘specialised killers’ toward ‘adaptive therapeutic systems’ capable of coordinated, multi‐layered control. Next‐generation CAR‐T cells should embody three core capabilities: (i) precise spatiotemporal regulation of activation to maximise tumour cytotoxicity while minimising off‐tumour effects [34, 35, 46]; (ii) intrinsic metabolic and epigenetic resilience to withstand TME‐driven exhaustion and sustain durable memory states [47, 48, 49]; and (iii) the capacity to actively remodel the immune microenvironment, converting immunologically ‘cold’ tumours into contexts supportive of T‐cell activity [18, 46, 50, 51, 52, 53, 54, 55]. Critically, these three pillars should be implemented cohesively rather than in isolation. For example, a CAR‐T cell that combines logic‐gated activation with enhanced mitochondrial fitness and tools to degrade the extracellular matrix or counteract inhibitory signalling is far more likely to penetrate, persist within, and control solid tumours. Overcoming these bottlenecks necessitates a paradigm shift from single‐target cytotoxic agents to integrated therapeutic systems capable of multi‐layered regulation of activation, persistence, and payload delivery. The generational evolution of CAR architectures charts this transition from single‐signal designs to multi‐modal platforms (Figure 4).

This review uniquely synthesises recent advances through this integrative lens, analysing how emerging receptor designs, cellular reprogramming strategies, and microenvironment‐modulating approaches can be rationally combined to create next‐generation CAR‐T products. We further contrast this integrative framework with the limitations of single‐axis interventions that often incur unacceptable efficacy‐toxicity trade‐offs, and we highlight the translational challenges and design principles for unifying these capabilities into a single, clinically viable therapeutic entity.

## 2 Engineering Next‐Generation CAR‐T Cells for Solid Tumours

Effective treatment of solid tumours requires CAR‐T cells that are precisely activated, maintain function, and adapt to resistance. This section examines integrated advances—controllable receptors, epigenetic and metabolic reprogramming, and multi‐antigen targeting—needed to achieve these objectives.

### 2.1 Precision Activation and Logic‐Gated Control

**FIGURE 5:**
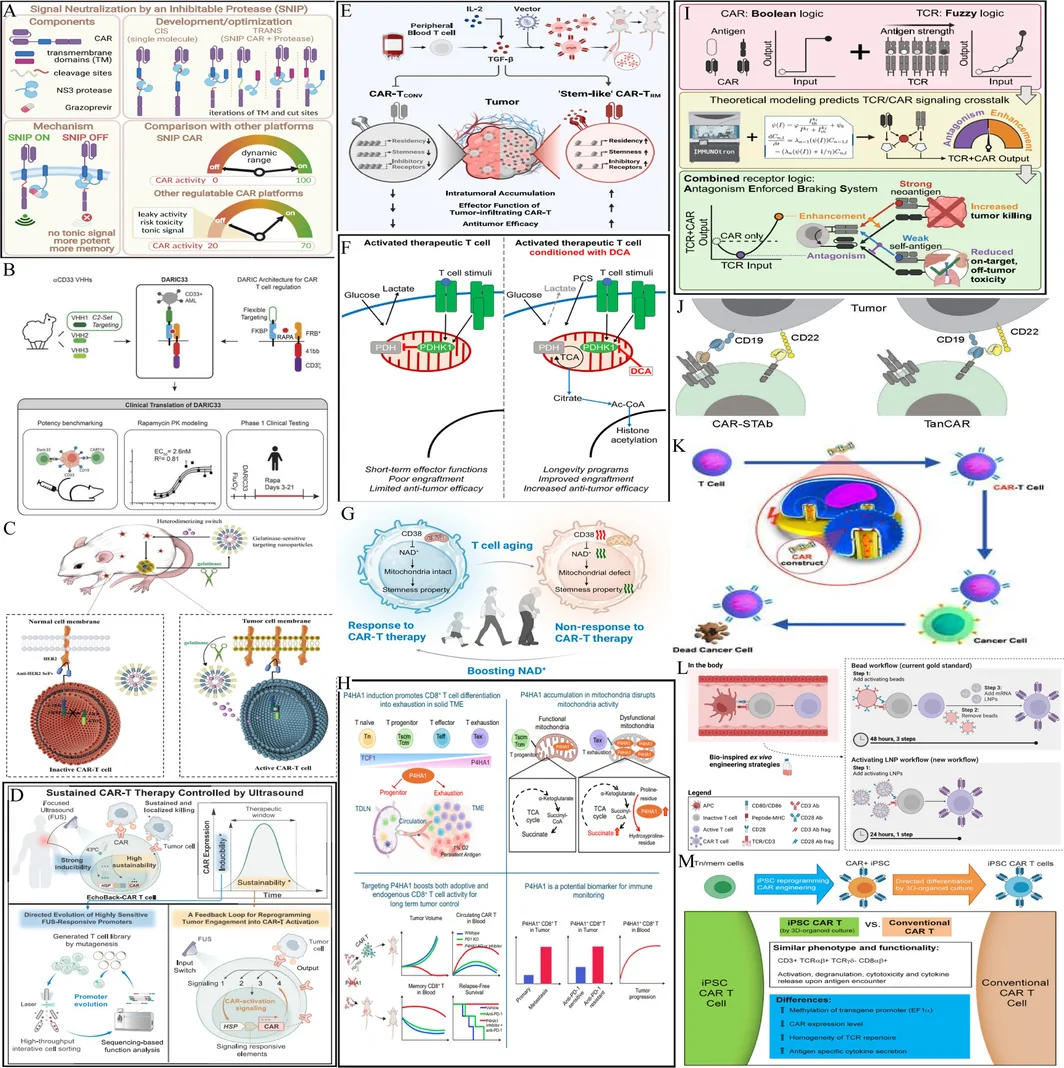
Integrative engineering strategies for optimizing CAR‐T cell specificity, metabolic fitness, and manufacturing efficiency. (A) Schematic illustration: Enhanced safety and efficacy of protease‐regulated CAR‐T cell receptors. Reproduced with permission [34]. Copyright from Elsevier Ltd., 2022. (B) Schematic illustration: Drug‐regulated CD33‐targeted CAR T cells control acute myeloid leukaemia (AML) using clinically optimized rapamycin dosing. Reproduced with permission [56]. Copyright from The American Society for Clinical Investigation (ASCI), 2024. (C) Schematic diagram illustrating the mechanism of tumour‐specific activation of switchable CAR‐T cells mediated by a gelatinase‐responsive NanoSwitch. Reproduced with permission [35]. Copyright from John Wiley and Sons, 2023. (D) Schematic illustration: Engineering sonogenetic EchoBack‐CAR T cells. Reproduced with permission [46]. Copyright from Elsevier Ltd., 2024. (E) Schematic illustration: Tissue‐resident memory CAR T cells with stem‐like characteristics display enhanced efficacy against solid and liquid tumours. Reproduced with permission [57]. Copyright from Elsevier Ltd., 2023. (F) Schematic illustration: Redirecting glucose flux during in vitro expansion generates epigenetically and metabolically superior T cells for cancer immunotherapy. Reproduced with permission [58]. Copyright from Elsevier Ltd., 2025. (G) Schematic illustration of the mechanism by which age‐related NAD^+^ decline impairs CAR‐T cell function. The diagram delineates how aging‐associated upregulation of the ecto‐enzyme CD38 accelerates NAD^+^ hydrolysis, resulting in a systemic NAD^+^ deficit that precipitates mitochondrial dysfunction and the erosion of T cell stemness properties. This metabolic deterioration compromises the therapeutic efficacy of CAR‐T cells in older individuals; however, as depicted, therapeutic intervention combining CD38 inhibition with NAD^+^ precursor supplementation can effectively reverse these deficits, restoring metabolic fitness and enhancing the antitumor potency of aged CAR‐T cells. Reproduced with permission [48]. Copyright from Elsevier Ltd., 2025. (H) Schematic illustration: Targeting prolyl 4‐hydroxylase subunit alpha 1 (P4HA1) promotes CD8^+^ T cell progenitor expansion toward immune memory and systemic anti‐tumour immunity. Reproduced with permission [59]. Copyright from Elsevier Ltd., 2025. (I) Schematic illustration: Engineering TCR‐controlled fuzzy logic into CAR T cells enhances therapeutic specificity. Reproduced with permission [60]. Copyright from Elsevier Ltd., 2025. (J) Schematic comparison of dual‐antigen targeting strategies designed to mitigate tumour immune escape. The illustration depicts two distinct engineering approaches for targeting CD19 and CD22 on tumour cells. The tandem chimeric antigen receptor (TanCAR) configuration (right) utilizes a single transmembrane chimeric receptor engineered with two distinct antigen‐binding domains arranged in tandem, allowing for the direct, simultaneous recognition of both surface antigens by the same CAR molecule. In contrast, the CAR‐STAb strategy (left) employs a combinatorial mechanism wherein T cells are engineered to express a specific CAR (targeting CD19) while concurrently secreting bispecific T‐cell engagers (TCEs) that independently bridge the T cell to a secondary tumour antigen (CD22), thereby creating a multi‐pronged immunological synapse. Reproduced with permission [61]. Copyright from BMJ Publishing Group Ltd., 2025. (K) Schematic illustration of the Electroactive Nanoinjection (ENI) platform for efficient CAR‐T cell engineering. The platform utilizes arrays of vertically configured conductive nanotubes to facilitate the non‐viral intracellular delivery of CAR constructs into primary human T cells. Upon the application of low‐voltage electrical pulses (10 V), the ENI platform induces transient membrane permeabilization at the nanotube‐cell interface, enabling precise nanoinjection of the genetic payload while maintaining high cell viability (> 90%). The resulting engineered CAR‐T cells demonstrate robust expression of the chimeric antigen receptor and exhibit potent cytotoxicity against target lymphoma cells. Reproduced with permission [62]. Copyright from John Wiley and Sons, 2023. (L) Biomimetic design and streamlined workflow of antigen‐presenting cell (APC)‐mimetic activating lipid nanoparticles (aLNPs). Drawing inspiration from physiological T cell activation—where APCs provide necessary primary (peptide—major histocompatibility complex (MHC)/TCR) and costimulatory (CD80/86‐CD28) signals—this platform integrates activation moieties directly onto the nanoparticle surface. In contrast to the current gold standard for ex vivo engineering, which necessitates a laborious multi‐step workflow involving magnetic bead‐mediated activation, bead removal, and subsequent mRNA LNP transfection over 48 h, the developed aLNP platform utilizes surface‐conjugated anti‐CD3 and anti‐CD28 antibody fragments to mimic APC function. This design enables the simultaneous activation and transfection of primary human T cells with CAR mRNA in a single, rapid step, effectively reducing the manufacturing timeline to 24 h by combining the immune‐stimulating properties of beads with the nucleic acid delivery capabilities of LNPs. Reproduced with permission [63]. Copyright from John Wiley and Sons, 2024. (M) Schematic illustration: 3D‐organoid culture supports differentiation of human CAR+ induced pluripotent stem cell (iPSCs) into highly functional CAR T cells. Reproduced with permission [64]. Copyright from Elsevier Ltd., 2022.

A central challenge in solid tumour CAR‐T therapy is achieving tumour‐restricted activity while simultaneously limiting on‐target/off‐tumour toxicity. One promising approach has been the development of controllable receptor systems with spatiotemporal precision. Pharmacologically regulated platforms offer external control over CAR‐T activity. For instance, the protease‐sensitive, neutralisable inhibitory circuit CAR (SNIP CAR) system utilises U.S. Food and Drug Administration (FDA)‐approved protease inhibitors to reversibly modulate CAR activation, thereby reducing T‐cell exhaustion and enhancing stemness compared with conventional CARs [34] (Figure 5A). Another approach, the chemical dimerisation of CD33 (DARIC33) platform, employs a rapamycin‐dependent split architecture to achieve tight control over cytokine release and cytotoxicity against CD33^+^ AML cells while sparing CD34^+^ haematopoietic progenitors, a strategy that has advanced to a phase I trial in paediatric AML [56] (Figure 5B). Beyond drug‐dependent control, stimuli‐responsive designs provide finer spatial and temporal regulation. NanoSwitch technology leveraged gelatinase‐responsive nanoparticles to selectively activate CARs within the TME, ensuring sustained CAR activity and curtailing systemic cytokine release [35] (Figure 5C). EchoBack‐CARs integrated heat shock promoters with feedback loops and achieved tumour clearance after brief focused ultrasound exposure; single‐cell RNA sequencing confirmed reduced exhaustion signatures in glioblastoma models [46] (Figure 5D). Together, these systems defined a therapeutic window for safe and effective tumour control.

Logic‐gated receptor architectures offer a particularly powerful strategy for refining specificity by introducing conditional antigen recognition. Protein‐split CARs implement an AND gate, separating antigen‐binding and signalling domains that only reconstitute upon engagement with dual antigens, thereby minimising off‐tumour effects [65]. A universal bispecific CAR platform extended this concept by using small molecule dimerisers to enforce dual‐antigen dependency, protecting normal tissues that express only a single antigen in leukaemia and solid tumour models [66]. Co‐expression of intracellular checkpoints such as SOCS1 within CAR‐T cells provided an additional means to mitigate toxicity, reducing CRS‐like manifestations by lowering inflammatory cytokines—a 70% decrease in IL‐6 and a 65% reduction in IFN‐γ—while preserving antitumour activity and improving the therapeutic index [67]. Control over the timing and location of activation is complemented by strategies that strengthen intrinsic potency and persistence.

Despite their elegant design, logic‐gated systems face significant translational hurdles that must be carefully addressed. Their efficacy relies on the stable and co‐localised expression of both antigens on every tumour cell, a requirement frequently compromised by the profound intra‐tumoural heterogeneity characteristic of human cancers. Antigen loss or downregulation of either target can render the logic gate ineffective, leading to therapeutic escape. Furthermore, the introduction of complex genetic circuits increases the genetic payload size, potentially impacting viral transduction efficiency and long‐term transgene stability. There is also a risk of ‘leaky’ basal signalling or unintended activation by physiological stimuli, which could lead to off‐tumour toxicity. The pharmacokinetics and optimal dosing of exogenous inducer molecules add another layer of clinical complexity. Therefore, while logic‐gating represents a paradigm shift in specificity, its success will depend on overcoming challenges related to tumour biology heterogeneity, genetic circuit reliability, and practical clinical delivery.

Recent advances continue to refine the specificity and programmability of logic‐gated systems. Fully human single‐chain variable fragments (scFvs) have been developed to mitigate immunogenicity risks associated with murine‐derived binders. For instance, a novel fully human anti‐B7‐H3 scFv (Y111) was generated via phage display and incorporated into a CAR; this construct demonstrated superior antitumour activity and persistence compared to CARs derived from traditional murine antibodies (376.96 and MGA271) in preclinical models of pancreatic cancer, neuroblastoma, and glioblastoma, highlighting the importance of binder humanisation for clinical translation [68]. Beyond dual‐antigen recognition, strategies are emerging to address glycan‐mediated immune evasion, a tumour‐intrinsic resistance mechanism. In classical Hodgkin lymphoma, the N‐glycosylation of CD30 at specific sites was found to shield the antigen from CAR‐T cell recognition. Pharmacological inhibition of glycosphingolipid synthesis with eliglustat, an FDA‐approved drug, trimmed terminal sialic acids from CD30 glycans, thereby enhancing the accessibility and cytotoxicity of CD30‐targeted CAR‐T cells and the antibody‐drug conjugate brentuximab vedotin, presenting a novel ‘glyco‐immunotherapy’ combination strategy [69]. Furthermore, the integration of artificial intelligence (AI) is beginning to guide the design of more sophisticated, multi‐input sensory CAR systems, moving beyond simple AND gates toward receptors capable of processing complex tumour microenvironmental signals. For instance, machine learning (ML) models can analyse vast proteomic and transcriptomic datasets to predict optimal antigen pairings for logic gates, minimising the risk of off‐tumour recognition due to heterogeneity [70]. More broadly, the application of automated machine learning (AutoML) with interpretable frameworks is emerging as a powerful tool in biomedical research. In the context of CAR design, AutoML can automate the complex process of feature engineering and model selection from high‐dimensional patient data, identifying critical biomarkers that predict response or resistance. Crucially, studies in other healthcare domains have demonstrated that AutoML systems equipped with explanation capabilities can generate high‐quality, interpretable models, thereby building clinician trust and facilitating deployment in complex clinical settings [71]. This paradigm could be directly translated to CAR‐T therapy for predicting patient‐specific toxicity risks or optimising manufacturing parameters, moving beyond black‐box predictions to generate actionable, mechanistic insights. This computational approach is critical to navigate the vast design space of synthetic receptors, ensuring that engineered circuits are not only elegant in theory but also robust and predictable in the heterogeneous biological reality of human tumours.

The clinical translation of logic‐gated and pharmacologically regulated CAR systems is progressing but remains at an early stage. While the DARIC33 platform (rapamycin‐dependent) has entered a phase I trial for paediatric AML (NCT05105152), and several synNotch‐based AND‐gate CAR constructs (e.g., targeting EGFRvIII/IL13Rα2 in glioblastoma, NCT05627323) are under evaluation, their application in solid tumours remains in its infancy. Recent clinical efforts have tested bivalent CAR‐T cells targeting EGFR and IL13Rα2 (CART‐EGFR‐IL13Rα2) via intrathecal delivery for recurrent glioblastoma. While early reductions in tumour enhancement were observed, dose‐limiting neurotoxicity (consistent with ICANS) was noted, and no objective radiographic responses were achieved in the first six patients, highlighting the safety and efficacy hurdles even with dual‐targeting approaches in the CNS [72]. For targets like B7‐H3, a first‐in‐human phase I trial of systemically administered B7‐H3 CAR‐T cells in paediatric/young adult solid tumours showed limited antitumour activity and poor expansion after the first infusion, though a second infusion in one patient led to marked CAR‐T expansion, an objective response, and concomitant cytokine release syndrome and transaminitis, illustrating the delicate balance between achieving sufficient expansion for efficacy and managing toxicity [73]. These early clinical experiences underscore the significant translational hurdles: the biological assumption of stable, co‐localised dual‐antigen expression is often violated by profound intra‐tumoural heterogeneity in human cancers; manufacturing complexity increases with multi‐component circuits; and the pharmacokinetics of exogenous inducers add clinical complexity. Therefore, while these systems offer a paradigm shift in specificity, their successful clinical translation will depend not only on elegant design but also on robust validation in patient‐derived models, scalable good manufacturing practice (GMP)‐compliant manufacturing, and carefully designed clinical trials that incorporate robust biomarker strategies.

### 2.2 Epigenetic and Metabolic Reprogramming

While precise activation governs the initiation of CAR‐T‐cell responses, sustained efficacy in solid tumours ultimately depends on the ability of CAR‐T cells to persist and function within a nutrient‐poor, hypoxic, and acidic TME. Intrinsic programming—spanning epigenetic and metabolic interventions—is central to preventing exhaustion and sustaining long‐term control. Epigenetic modulation has improved durability in several models. FOXO1 overexpression increased chromatin accessibility at its binding sites and induced a memory‐associated transcriptional program, which preserved function and metabolic integrity under chronic antigen exposure and yielded superior in vivo persistence [49]. Complementary gains were achieved by combining Runx3 overexpression with AKT inhibition during manufacturing, producing cells with hybrid central memory (Tcm) and tissue‐resident memory (TRM) attributes that exhibited extended lifespan and resistance to exhaustion [74]. Transient exposure to TGF‐β during culture augmented TRM signatures; TGF‐β‐conditioned CAR‐TRM cells showed enhanced effector cytokine production and antitumour activity across malignancies [57] (Figure 5E). Pharmacologic approaches, including histone deacetylase (HDAC) inhibition with agents such as entinostat, promoted memory differentiation, supported mitochondrial metabolism, and improved persistence upon antigen rechallenge [75].

While epigenetic interventions show great promise preclinically for enhancing CAR‐T cell fitness, translating them into robust and scalable manufacturing processes poses distinct challenges. The primary hurdle lies in the temporal control and specificity of the epigenetic modulation. Pharmacological approaches, such as adding vitamin C, butyrate, or HDAC inhibitors during ex vivo culture, are relatively straightforward to integrate into existing manufacturing protocols. However, achieving consistent and durable effects across cell batches requires precise optimisation of concentration, timing, and duration of exposure, which adds a layer of process complexity. More sophisticated genetic engineering strategies—like the stable overexpression of transcription factors or the use of inducible systems to control epigenetic modifiers—introduce additional manufacturing and safety considerations. These include increased genetic payload size, potential for insertional mutagenesis, and the risk of unintended, off‐target epigenetic changes that could affect long‐term cell behaviour or oncogenic potential. Furthermore, the combination of multiple epigenetic edits with the core CAR construct, a likely requirement for maximal benefit, exacerbates these challenges. It necessitates more complex vector design, raises the bar for quality control, and complicates regulatory approval pathways. Therefore, advancing epigenetic reprogramming from bench to bedside will require not only a deeper biological understanding of the optimal targets but also parallel innovations in vectorology, gene‐editing safety, and streamlined manufacturing workflows capable of robustly producing these multi‐engineered cell products.

Metabolic reprogramming is likewise required to maintain fitness in hostile conditions. Shifting hyper‐glycolytic, in vitro–expanded T cells toward oxidative phosphorylation (OXPHOS) using dichloroacetate (DCA)—a pyruvate dehydrogenase kinase inhibitor—improved mitochondrial performance, increased metabolic flexibility, reduced glucose dependence, and enhanced in vivo antitumour activity [58] (Figure 5F). Age‐related decline in function was linked to reduced NAD^+^ levels driven by elevated CD38, resulting in mitochondrial impairment and loss of stemness; suppressing CD38 or replenishing NAD^+^ with precursors restored activity in aged CAR‐T cells and strengthened therapeutic potential [48] (Figure 5G). The mitochondrial enzyme prolyl 4‐hydroxylase subunit alpha 1 (P4HA1) served as a metabolic–immune checkpoint; it accumulated in exhausted CD8^+^ T cells, disrupted the tricarboxylic acid (TCA) cycle, and promoted mitochondrial dysfunction. Inhibition of P4HA1 expanded precursor CD8^+^ T cells and augmented systemic antitumour immunity, with P4HA1^+^ CD8^+^ T cells marking cancer progression [59] (Figure 5H). Intermittent hypoxia further exacerbated mitochondrial dysfunction and reactive oxygen species accumulation, undermining metabolic adaptability and effector function [76]. Beyond sustaining fitness, CAR‐T cells can be configured to exploit context‐specific cytotoxic pathways. CAR‐T‐derived IFN‐γ downregulated tumour glutathione peroxidase 4 (GPX4), sensitising tumour cells to ferroptosis—an iron‐dependent, lipid peroxidation–driven form of death—which complemented perforin/granzyme‐mediated killing, particularly in immunologically ‘cold’ settings [77].

### 2.3 Overcoming Antigen Escape and Resistance

Despite significant advances in precision and resilience, therapeutic efficacy is frequently undermined by tumour adaptation, primarily through antigen escape and intrinsic signalling defects. Among these, antigen loss or downregulation represents a frequent resistance mechanism. In multiple myeloma, relapse after BCMA‐targeted CAR‐T therapy was associated with homozygous deletion of TNFRSF17 (BCMA) in 33% of relapsed cases, facilitating selection of BCMA‐negative clones [78]. Resistance in high‐grade B‐cell lymphomas was multifactorial, involving antigen loss, clonal evolution, and T‐cell dysfunction [79]. In metastatic castration‐resistant prostate cancer (mCRPC), STEAP1 downregulation occurred in 17%–30% of cases after CAR‐T infusion despite high baseline expression [18, 80].

Engineering strategies that incorporate multiple target antigens can effectively mitigate immune escape. Tandem CD19/CD20 CARs driven by EF‐1α bidirectional promoters increased cytotoxicity and improved survival in lymphoma models compared with single‐target constructs [81]. This principle of multi‐targeting is especially critical for virus‐associated tumours, where the high mutational rate of viral genomes can lead to rapid emergence of escape variants. Designing a bispecific or tandem CAR against two conserved epitopes of an oncogenic virus represents a rational strategy to pre‐empt this resistance mechanism. Receptor cross‐talk further refined precision; dual TCR/CAR‐T cells used differential TCR signal strength to modulate CAR activation [60] (Figure 5I). Secretory platforms recruited bystander immunity. CAR‐secreted T‐cell engaging antibody (CAR‐STAb) T cells produced CD19 T‐cell engagers that selectively targeted CD22, promoting endogenous T‐cell involvement and limiting antigen loss escape [61] (Figure 5J). An mRNA/nanobody co‐delivery platform generated transient dual‐specificity T cells (CAR + engager), balancing potency with safety [82]. Advances in delivery and manufacturing have also supported these approaches. Non‐viral systems improved scalability and safety; an electroactive nano‐injection (ENI) platform employing vertically aligned nanotubes for localised electroporation increased CAR transfection efficiency threefold while maintaining over 90% viability at low voltages [62] (Figure 5K). Antigen‐presenting cell–mimetic lipid nanoparticles (aLNPs) co‐delivered CD3/CD28 antibodies and CAR mRNA, enabling single‐step activation and transfection and achieving in vivo activity comparable to bead‐activated cells [63] (Figure 5L).

These advances—spanning controllable receptors, CRISPR‐mediated functional enhancement, and dual‐targeting synapse design—define a multidimensional engineering trajectory toward CAR‐T cells with context‐sensitive control, sustained function, and long‐term durability. This framework, summarised in Table 1, supports the development of cellular therapeutics that can adapt within the TME. Continued refinement, including induced pluripotent stem cell (iPSC)–derived CAR‐T cells with uniform TCR clonality and potent antitumour activity, offers a feasible off‐the‐shelf path for allogeneic use [64, 84] (Figure 5M). Co‐expression of TAPi and class II transactivator (CIITA)‐targeted shRNA to downregulate major histocompatibility complex (MHC) I/II reduced immunogenicity and the risk of host rejection—an important consideration given documented anti‐CAR responses after repeated dosing—and expands the potential utility of CAR‐T therapies. Integration of precision activation, intrinsic resilience, and multi‐targeting capabilities is necessary to address the sequential barriers imposed by the solid TME and advance CAR‐T cells toward durable efficacy in solid malignancies.

**TABLE 1:** Engineered CAR‐T platforms: Mechanisms and therapeutic applications.

CAR‐T type	Mechanism	Function	Cancer immunotherapy application	References
SNIP CAR	Protease inhibitor‐regulated CAR activation	Safety switch controllable; reduced T‐cell exhaustion	Solid tumours with mitigated on‐target/off‐tumour toxicity	[34]
DARIC33 CAR	Rapamycin‐dependent split CAR architecture	Pharmacologically controlled cytotoxicity; haematopoietic stem cell (HSC) sparing	Relapsed/refractory AML	[56]
NanoSwitch CAR	Gelatinase‐responsive nanoparticle‐mediated dimeriser delivery	Tumour‐specific activation; attenuated cytokine release	Solid tumours with enhanced safety profile	[35]
EchoBack CAR	Ultrasound‐inducible HSF1 promoter with positive feedback loop	Spatiotemporally controlled activation; reduced exhaustion	Glioblastoma with minimised off‐target toxicity	[46]
Split AND‐gate CAR	Protein‐fragment complementation requiring dual antigens	Enhanced tumour selectivity; reduced off‐tumour toxicity	Heterogeneous solid tumours	[65]
Universal bispecific CAR	Small molecule AND dual‐antigen gated activation	Multilayered specificity; normal tissue protection	Solid tumours with stringent safety control	[66]
SOCS1‐modified CAR‐T	SOCS1 co‐expression to attenuate cytokine signalling	CRS mitigation without compromising efficacy	Broad applications with improved therapeutic index	[67]
FOXO1‐OE CAR‐T	FOXO1‐driven memory programming	Chromatin accessibility at memory loci; metabolic fitness preservation	Chronic antigen exposure scenarios	[49]
Runx3‐OE/AKT‐i CAR‐T	Runx3 overexpression and AKT inhibition	Dual central/tissue‐resident memory (TRM) phenotype; enhanced tumour homing	Solid tumours resistant to PD‐1 blockade	[74]
TGF‐β‐primed CAR‐TRM	TGF‐β‐induced TRM differentiation	Stem‐like properties (TCF1andIL‐7Rand); resistant to TME dysfunction	Solid/liquid tumours with immunosuppressive TME	[57]
HDACi‐modulated CAR‐T	Entinostat‐mediated epigenetic reprogramming	Mitochondrial metabolism enhancement; anti‐apoptotic capacity	Repeated antigen challenge settings	[75]
DCA‐reprogrammed CAR‐T	Pyruvate dehydrogenase kinase inhibition promotes oxidative phosphorylation (OXPHOS) shift	Reduced glucose dependence; metabolic flexibility and stemness	Glucose‐addicted malignancies	[58]
NAD^+^‐restored geriatric CAR‐T	CD38 inhibition and NAD^+^ precursor supplementation	Mitochondrial function rescue; rejuvenated stemness	Elderly patients (> 65 years)	[48]
P4HA1‐targeted T cells	Reversal of P4HA1‐mediated TCA cycle disruption	Precursor CD8and T‐cell expansion; systemic antitumour immunity restoration	Solid tumours with dysfunctional TILs	[59]
Intermittent hypoxia‐exposed CAR‐T	Hypoxia‐induced mitochondrial dysfunction and reactive oxygen species (ROS) accumulation	Impaired cytotoxicity and proliferation	Patients with comorbidities (e.g., sleep apnea)	[76]
Ferroptosis‐enhanced CAR‐T	IFN‐γ‐mediated GPX4 downregulation	Tumour cell ferroptosis induction (contact‐independent killing)	Immunologically ‘cold’ solid tumours	[77]
		Dual cytotoxicity/ferroptosis induction	Solid tumours with limited direct contact	
TNFRSF17‐edited MM therapy	Monitoring 16p deletions pre‐therapy	Identification of high‐risk relapse patients	MM with chr16 monosomy	[78]
Multitargeted high‐grade B‐cell lymphoma (HGBL) approach	Addressing clonal evolution and T‐cell defects	Prevention of antigen escape in TP53‐mutated tumours	Aggressive HGBL‐TH lymphoma	[79]
STEAP1 CAR‐T and IL‐12	IL‐12‐mediated TME remodelling	Overcame STEAP1 escape; activated endogenous immunity	mCRPC (87.7% STEAP1and tumours)	[18]
TCR/CAR integrated T cells	Engineered TCR‐CAR cross‐regulation (weak TCR → CAR inhibition)	Context‐dependent activation; reduced on‐target/off‐tumour toxicity	Solid tumours with improved specificity	[60]
CAR‐STAb‐T cells	CD22‐CAR secreting CD19 T‐cell engagers	Bystander T‐cell recruitment; mitigation of antigen loss relapse	B‐ALL with superior efficacy vs. dual‐CAR	[61]
LNP‐mRNA/nanobody CAR‐T	Co‐delivery of CAR mRNA and bispecific nanobodies via LNPs	Rapid generation of dual‐specificity T cells; transient expression	B‐cell malignancies with adaptable targeting	[82]
TAPi/shCIITA CAR‐T	Dual knockdown of MHC I/II via TAP inhibitor and CIITA shRNA	Reduced immunogenicity for autologous/allogeneic use	Broad applications with mitigated host rejection	[83]

A transformative shift is underway from ex vivo manufacturing to in vivo generation of CAR‐T cells, aiming to directly reprogram endogenous T cells within patients. This approach could dramatically improve accessibility and reduce costs [85]. Key to this strategy are advanced delivery platforms. Targeted lipid nanoparticles (tLNPs) have shown particular promise. Systematic comparison of T‐cell receptor‐targeting moieties revealed that CD7‐targeted tLNPs achieved the highest mRNA delivery efficiency to human T cells, a property linked more to receptor internalisation kinetics than abundance [86]. These CD7‐tLNPs successfully generated functional anti‐CD20 CAR‐T cells in vivo in humanised mouse models. Beyond viral vectors, bacterial biomaterials, such as engineered outer membrane vesicles (OMVs), are being explored as versatile delivery and immunomodulatory agents. One platform, BROAD‐CAR, utilised OMVs modified to express an anti‐PD‐L1 nanobody and loaded with plasmids encoding a tumour‐associated antigen. This system not only blocked PD‐1/PD‐L1 interaction to reverse immunosuppression but also facilitated in situ antigen painting of tumours, enabling CAR‐T cells to target antigen‐heterogeneous and antigen‐negative tumours, thereby inhibiting recurrence and metastasis in breast cancer models [87]. Bacterial biomaterials can also be engineered to remodel the TME and enhance the infiltration and function of adoptively transferred CAR‐T cells [88].

## 3 Mapping the Target Landscape for CAR‐T Therapy

The successful transition from engineering innovation to clinical application critically depends on selecting antigens that discriminate malignant from normal tissues with sufficient precision. While the success of CD19 and BCMA‐targeted CAR‐T cells in hematologic malignancies established the foundational importance of target selection, the challenges in solid tumours are distinct and more complex. This section first outlines the core criteria for target selection—tumour specificity, functional essentiality, and therapeutic window—drawing lessons from both hematologic and solid tumour experiences. We then focus our analysis on two key domains for solid tumours: (1) solid tumour–restricted and associated antigens, and (2) pan‐cancer and neoantigen targets. The lessons learned from targeting hematologic antigens, particularly regarding antigen escape and on‐target/off‐tumour toxicity, directly inform the more stringent requirements for solid tumour target validation [89]. A systematic overview of promising solid tumour targets, their biological rationale, and associated challenges is summarised in Table 2. In addition to these conventional TAAs, a distinct class of targets exists in virus‐associated malignancies: oncogenic viral antigens. These foreign proteins, essential for tumourigenesis in 10%–20% of human cancers, offer a unique opportunity for highly specific CAR‐T cell therapy with a theoretically negligible risk of on‐target/off‐tumour toxicity [120].

**TABLE 2:** Molecular targets for CAR‐T therapy in solid and hematologic tumours.

CAR‐T type	Mechanism	Function	Cancer immunotherapy application	References
B7‐H3 CAR‐T	Targeting transmembrane immunomodulator B7‐H3 (CD276)	Induced tumour regression in orthotopic models	Paediatric brain tumours (medulloblastoma, ependymoma)	[90]
B7‐H3 CAR‐T and PHCN	Catalytic nanoparticle‐mediated TME disruption	Enhanced CAR‐T infiltration and activation	NSCLC with synergistic tumour eradication	[91]
αvβ3 CAR‐T	Targeting integrin complex overexpressed in gliomas	Specific tumour clearance and long‐term immunological memory	DIPG and glioblastoma	[92]
CD317 CAR‐T	Targeting transmembrane protein CD317	Specific glioma cell lysis and survival extension	Glioblastoma	[93]
GPC2 CAR‐T (CT3‐based)	Targeting tumour‐specific exons 3/7–10 identified by RNA‐seq	Specific killing of neuroblastoma cells	Neuroblastoma with minimal on‐target/off‐tumour toxicity	[94]
PTK7 CAR‐T	Surface glycoproteomics‐identified target	Eradicated metastatic deposits without toxicity	Chemotherapy‐resistant neuroblastoma	[95]
EphA3 CAR‐T	Targeting overexpressed receptor in high‐grade gliomas	Sustained complete remission (> 6 months) and rechallenge protection	Glioblastoma/diffuse midline glioma	[96]
EDA CAR‐T	Targeting fibronectin splice variant in stroma/vasculature	Dual cytotoxic/anti‐angiogenic activity and bystander killing	Broad solid tumours (validated in HCC PDX)	[97]
PS‐CAR‐T	Targeting immunoregulatory lipid on tumour/vasculature	Dual tumour cell killing and vascular disruption	Broad solid tumours (anti‐angiogenic effect)	[98]
MSLN CAR‐T and alkalinisation	pH‐dependent antigen modulation	Restored cytotoxicity via TME pH normalisation	TNBC with heterogeneous MSLN expression	[99]
AXL CAR‐T and MWA	Targeting receptor tyrosine kinase AXL	Enhanced infiltration and mitochondrial metabolism post‐MWA	NSCLC metastases	[100]
CD133 CAR‐T	Targeting cancer stem cell marker	Preclinical efficacy against lung/colon brain metastases	Brain metastases (considering heterogeneity/CD133‐stem cells)	[101]
nfP2X7 CAR‐T	Targeting stress‐induced ectodomain	Broad cytotoxicity across 24 carcinoma lines	Pan‐carcinoma (breast/prostate PDX models)	[102]
MICA/B α3 CAR‐T	Targeting non‐cleavable α3 domain	Resisted soluble immunosuppression	MICA/B‐shedding solid tumours	[103]
GRP78 CAR‐T	Targeting stress chaperone surface expression	Context‐dependent efficacy affected by CAR‐T activation state	GBM, DIPG, osteosarcoma, TNBC	[104]
GPA33 CAR‐T	Targeting basolateral tumour expression	Specific killing of CRC PDX cells	Colorectal cancer (apical‐basolateral differential)	[105]
CLDN18.2 CAR‐T	Targeting tight junction protein	Complete responses in heavily pretreated patients	Gastric/pancreatic cancer (Phase I trial)	[106]
CD112RIVE CAR‐T	Structure‐guided high‐affinity CAR design	Enhanced T‐cell activation against low‐density targets	CD112and TNBC	[107]
PAP coupled CAR‐T	Coupled CAR platform for antigen‐specific expansion	Superior efficacy vs. conventional CAR‐T	Advanced prostate cancer	[108]
CD33 (CD28) CAR‐T	CD28‐costimulated construct for superior proliferation	Selected for clinical translation (lintuzumab‐CD28ζ)	Paediatric R/R AML (Phase I trial)	[109]
CD33 (membrane‐proximal) CAR‐T	High‐affinity binder to IgC domain	Superior efficacy in low antigen density models	AML (outperformed distal epitope‐targeting CAR)	[110]
AdCAR‐T (CD33/CD38/etc.)	Aptamer‐mediated combinatorial targeting	Overcame intratumoural heterogeneity in PDX models	Heterogeneous AML	[111]
CD27ζ CAR‐T	Targeting CD70 ligand for enhanced proliferation	AML cell elimination without HSC toxicity	CD70and AML	[112]
CLL1 CAR‐T	Targeting LSC‐associated antigen	50% MRD‐CR in paediatric R/R AML	Relapsed/refractory AML	[113]
CLL1 CAR‐T (JMML)	Multimodal omics‐identified LSC target	Reduction of leukaemia stem cells in PDX	Juvenile myelomonocytic leukaemia	[114]
CD72 (H24) CAR‐T	Humanised nanobody with optimised framework	Enhanced cytotoxicity against CD19‐CAR relapsed samples	Refractory B‐cell malignancies	[115]
LILRB1 CAR‐T	Targeting immune checkpoint on B‐ALL/B‐NHL	Activity against CD19‐CAR resistant clones	Salvage therapy for CD19‐/CD20‐immunotherapy failures	[116]
SLC3A2 CAR‐T	Targeting cystine transporter on tumour surface	Tumour growth inhibition in xenografts	Broad cancers (metabolic vulnerability)	[117]
Anxa CARLow‐T	Low‐density CAR to avoid fratricide	Achieved antitumour activity post‐suicide mitigation	PS‐exposing tumours	[98]
mKRAS NeoCAR (iIL‐12)	TCR knockout neoantigen targeting and inducible IL‐12	Enhanced antigen presentation and safety	KRAS G12V‐mutated lung/pancreatic/renal cancers	[118]
TCR‐T (splicing‐derived)	Targeting recurrent frameshift neoantigens	Selective killing of spliceosome‐mutant cells	AML/MDS with splicing factor mutations	[119]

### 3.1 Solid Tumour‐Specific Antigens

**FIGURE 6:**
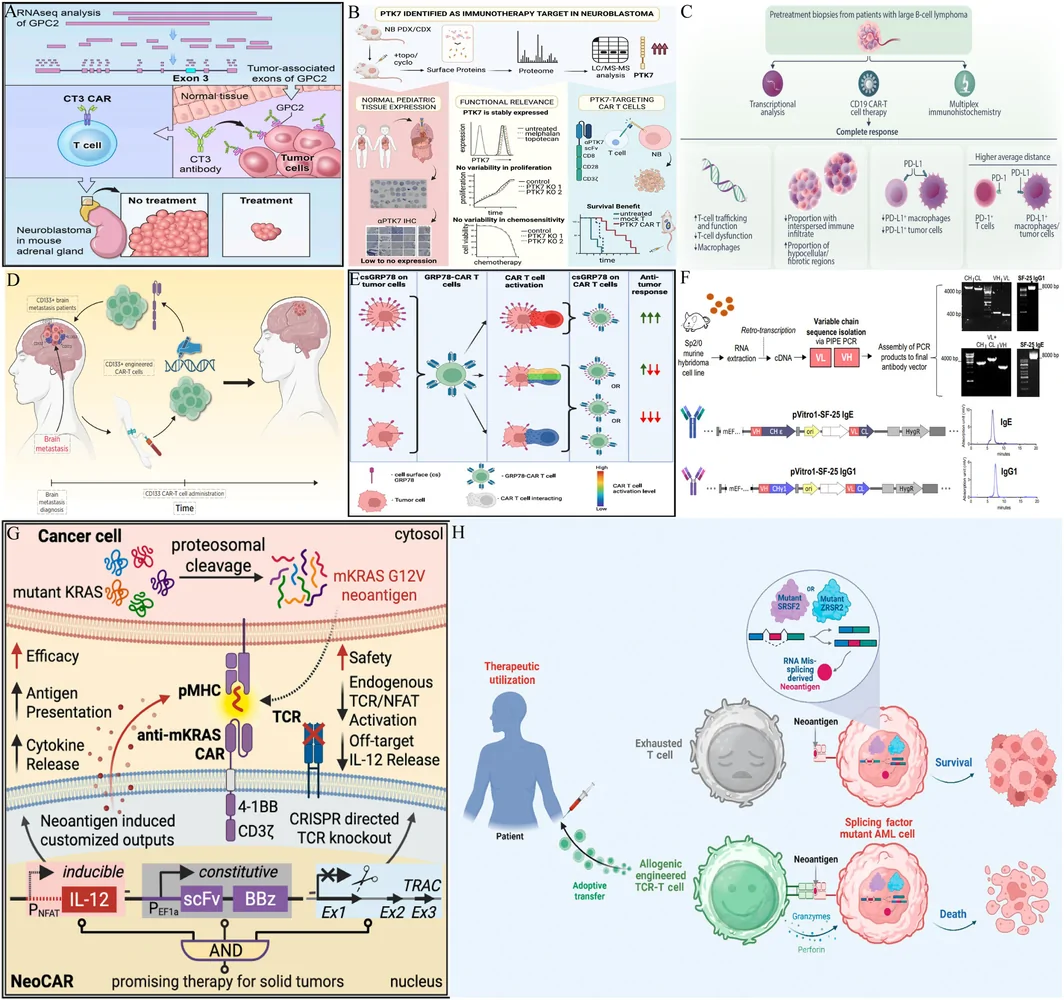
Novel target identification and precision engineering strategies for advancing CAR‐T and TCR‐T immunotherapies in oncology. (A) Schematic illustration: CAR T cells targeting tumour‐associated exons of glypican 2 (GPC2) regress neuroblastoma in mice. Reproduced with permission [94]. Copyright from Elsevier Ltd., 2021. (B) Schematic illustration: Identification and targeting of protein tyrosine kinase 7 (PTK7) as an immunotherapy candidate for neuroblastoma. Reproduced with permission [95]. Copyright from Elsevier Ltd., 2023. (C) Schematic illustration: PD‐L1^+^ macrophage and tumour cell abundance and proximity to T cells in the pretreatment large B‐cell lymphoma microenvironment impact CD19 CAR‐T cell immunotherapy efficacy. Reproduced with permission [96]. Copyright from John Wiley and Sons, 2024. (D) Schematic representation of the therapeutic strategy utilizing CD133‐targeted CAR‐T cells for the treatment of brain metastases. Given the elevated expression of the cancer stem cell antigen CD133 in metastatic brain lesions, this approach outlines a personalized immunotherapy workflow wherein autologous T cells are harvested following diagnosis, genetically engineered to express anti‐CD133 chimeric antigen receptors, and adoptively transferred back into the patient to specifically target and eradicate CD133‐positive tumour cells. Reproduced with permission [101]. Copyright from American Association for Cancer Research, 2024. (E) Schematic illustration: GRP78‐CAR T cell effector function against solid and brain tumours is controlled by GRP78 expression on T cells. Reproduced with permission [104]. Copyright from Elsevier Ltd., 2023. (F) Engineering and testing of SF‐25 chimeric antibodies. This schematic illustrates the complete workflow for engineering and characterizing SF‐25 antibodies fused with human IgG1 or IgE Fc regions. The process outlines the isolation of SF‐25 variable regions, validated by representative agarose gel images of PCR products, followed by the assembly of final sequence constructs within pVitro1 expression vectors. These vectors were subsequently utilized to produce full‐length chimeric SF‐25 IgE and IgG1 antibodies, the quality and integrity of which were assessed using high‐performance liquid chromatography (HPLC) elution profiles. Reproduced with permission [117]. Copyright from BMJ Publishing Group Ltd., 2021. (G) Mutant KRAS peptide targeted CAR‐T cells engineered for cancer therapy. Reproduced with permission [118]. Copyright from Elsevier Ltd., 2025. (H) Schematic illustration of targeted immunotherapy utilizing engineered TCR‐T cells against RNA mis‐splicing‐derived neoantigens in acute myeloid leukaemia (AML). Splicing factor mutations generate recurrent neoantigens via RNA mis‐splicing, which typically render endogenous T cells functionally exhausted. By contrast, the adoptive transfer of high‐affinity engineered TCR‐T cells bypasses this immune dysfunction, specifically recognizing these mis‐splicing‐derived targets and effectively eliminating leukaemic cells via perforin and granzyme‐mediated cytotoxicity. Reproduced with permission [119]. Copyright from Elsevier Ltd., 2025.

Central nervous system (CNS) tumours require targets with restricted physiological expression and functional relevance. B7‐H3 (CD276) is markedly overexpressed in paediatric medulloblastoma and ependymoma relative to baseline levels. B7‐H3–CAR‐T cells induced tumour regression in orthotopic models after systemic or localised delivery, with efficacy further augmented by catalytic nanoparticles (PHCN) that disrupted the extracellular matrix and improved T‐cell infiltration [90, 91]. The αvβ3 integrin complex showed high expression in diffuse intrinsic pontine glioma (DIPG) and glioblastoma with limited normal tissue distribution; targeting enabled complete tumour eradication and durable immunologic memory upon rechallenge [92]. Targeting CD317, which localises to glioma membranes, has shown promise, with CAR‐T cells demonstrating antitumour activity and prolonging survival in orthotopic models—findings corroborated by antigen‐silencing studies [93]. In paediatric neuroblastoma, epitope‐level precision was achieved with glypican‐2 (GPC2), whose cancer‐associated exons (3 and 7–10) are absent from normal tissues; high‐affinity CT3‐derived CAR‐T cells improved regression while reducing off‐tumour binding [94] (Figure 6A). PTK7 was consistently expressed after chemotherapy, enabling detection of metastatic deposits while sparing healthy paediatric tissues [95] (Figure 6B). EphA3 was enriched in aggressive gliomas; EphA3–CAR‐T cells showed strong in vitro cytotoxicity and sustained complete remissions in vivo exceeding 6 months post‐treatment [96] (Figure 6C).

Stromal and vascular antigens provide complementary intervention points. The fibronectin splice variant Extra Domain A (EDA), restricted to tumour stroma and neovasculature, increased lysis of EDA^+^ cells and enabled bystander killing of EDA− tumours via vascular disruption in immunocompetent models and hepatocellular carcinoma patient‐derived xenografts [97]. Phosphatidylserine (PS), aberrantly displayed on tumour and endothelial cells, emerged as a tractable target across indications; PS–CAR‐T cells exerted cytotoxic and anti‐angiogenic effects and suppressed growth in multiple solid tumours in vivo [98]. Microenvironmental acidity affected target display, as shown for mesothelin (MSLN) in triple‐negative breast cancer (TNBC), where low pH heightened antigen heterogeneity and limited efficacy; alkalinisation increased surface expression and CAR‐T cytotoxicity [99].

Metastasis‐associated antigens are under active investigation. STEAP1 exhibited broader expression than PSMA (87.7% in mCRPC vs. 60.5%), yet monotherapy was constrained by antigen escape; targeted IL‐12 delivery reconditioned the microenvironment, reduced exhaustion, and enhanced endogenous immunity to counter resistance [18, 80]. AXL, elevated in non‐small cell lung cancer metastases, produced partial regression alone, whereas microwave ablation (MWA) improved CAR‐T infiltration, persistence, and intratumoural metabolism [100]. CD133 (Prominin‐1), present on brain‐metastatic cancer stem cells, is accessible to CAR‐T cells across the blood–brain barrier, but heterogeneous expression and potential neural stem cell toxicity necessitate stromal modulation for safe application [101] (Figure 6D).

Efforts to identify universal solid tumour antigens aim to address inter‐ and intra‐tumoural heterogeneity. The nonfunctional P2X7 receptor (nfP2X7) was overexpressed in carcinomas and absent in healthy cells; targeting increased complete responses in breast and prostate patient‐derived xenografts without evident toxicity [102]. Focusing on the MICA/B α3 domain reduced proteolytic shedding, enabling CAR‐T cells to resist soluble suppression while preserving cytotoxicity against epithelial cancers [103]. Target modulation can be bidirectional; surface GRP78 was upregulated in solid and brain tumours, yet elevated GRP78 in T cells diminished efficacy, indicating a need to integrate metabolic controls [104] (Figure 6E).

Clinically validated targets mark key translational steps. Glycoprotein A33 (GPA33) showed consistent expression in colorectal cancer with apical localisation in normal tissue and basolateral localisation in tumours, supporting CAR‐T activity in patient‐derived xenografts [105]. Claudin 18.2 (CLDN18.2), prevalent in gastric and pancreatic cancers, yielded promising responses, including complete remissions, with manageable safety in phase I studies [106]. Claudin 18.2 (CLDN18.2), prevalent in gastric and pancreatic cancers, has yielded some of the most promising clinical signals in solid tumour CAR‐T therapy to date. Initial phase I studies of the autologous CLDN18.2‐targeted CAR‐T product CT041 (satricabtagene autoleucel) reported encouraging activity in heavily pretreated gastric/gastroesophageal junction (G/GEJ) cancer patients [10]. This promise was recently confirmed in a pivotal phase 2 randomised controlled trial (NCT04581473), where satri‐cel demonstrated a significant improvement in median progression‐free survival (3.25 vs. 1.77 months) compared to treatment of physician's choice in patients with CLDN18.2‐positive advanced G/GEJ cancer, representing a landmark as the first positive randomised trial of CAR‐T therapy in solid tumours [121]. In pancreatic cancer, a pooled analysis of phase I/Ib trials also showed signals of activity for CT041, with a disease control rate of 70.8% and a median overall survival of 10.0 months [122]. However, these studies also reveal critical challenges. Efficacy appears heterogeneous across patients, and factors such as antibiotic use for intercurrent infection have been associated with significantly reduced progression‐free and overall survival, an effect potentially mediated by gut microbiome alterations [123]. Furthermore, an elevated neutrophil‐to‐lymphocyte ratio (NLR) was prognostic for poorer outcomes, highlighting the impact of systemic inflammation and the immunosuppressive myeloid compartment on treatment success [124]. These clinical findings underscore that even for a promising target like CLDN18.2, patient selection, management of concomitant medications, and modulation of the host immune environment are crucial determinants of clinical benefit. Structural insights refined target engagement; delineation of the CD112–CD112R interaction enabled the directed evolution of high‐affinity CD112RIVE variants that enhanced activation against TNBC cells versus wild‐type receptors [107]. A ‘coupled CAR’ system for prostate‐specific PAP increased the frequency of antigen‐specific CAR‐T cells and improved efficacy compared with conventional designs, advancing outcomes in advanced prostate cancer [108].

For targets with ideal tumour specificity but intracellular localisation, TCR‐like CARs targeting peptide–MHC complexes offer a solution. Recent work has developed nanobody‐based TCR‐like CAR‐T cells targeting a PRAME‐derived peptide (PRAME425‐433) presented by HLA‐A02:01. PRAME is highly expressed in acute myeloid leukaemia (AML) but largely absent in normal tissues. These CAR‐T cells showed potent and specific anti‐leukaemic activity in vitro and in vivo against PRAME+/HLA‐A02:01^+^ AML cells, with negligible toxicity to normal haematopoietic stem and progenitor cells, presenting a promising strategy for targeting intracellular cancer‐testis antigens [125].

Persistent challenges include antigen loss (e.g., STEAP1), motivating dual‐targeting or escape‐preventive designs; limited infiltration into dense cores, necessitating improved delivery and microenvironmental modulation; and ongoing risks of on‐target/off‐tumour toxicity, which remain unresolved for certain targets such as CD133. Strategies combining dual‐antigen logic gates (TanCAR), TME‐responsive switches (protease‐cleavable linkers), and epigenetic/metabolic priming to stabilise antigen expression warrant further evaluation.

### 3.2 Hematologic Immune Checkpoint Targets

Whereas solid tumours pose microenvironmental barriers, hematologic malignancies require precise target selection that preserves haematopoiesis. In AML, CD33 is a principal target in which CAR architecture influences efficacy. CD28‐costimulated CD33 CARs exhibited greater antileukemic activity than 4‐1BB variants, associated with increased proliferation and cytokine release, supporting the clinical use of lintuzumab‐CD28ζ in paediatric relapsed/refractory AML [109]. Epitope positioning also mattered: membrane‐proximal CD33 intestinal‐type gastric cancer (IgC) domain binders (3P14HLh28Z) outperformed distal epitope CARs in low antigen–density models by stabilising synapses [110]. The Adapter CAR (AdCAR) platform used aptameric adaptors to address antigenic heterogeneity by redirecting to CD33, CD38, CD123, CD135, and CD371, achieving complete remissions in multiple patient‐derived xenografts and limiting monotherapy escape [111].

New targets have expanded the therapeutic window. CD70 showed selective expression on AML blasts and leukaemic stem cells but was absent on haematopoietic stem cells; CD27ζ CARs increased proliferation, whereas CD70 CARs maintained specific cytotoxicity without impairing haematopoietic stem cells [112]. CLL1, with expression restricted to leukaemic stem cells, enabled autologous CAR‐T cells to achieve MRD‐negative remissions in 50% of paediatric relapsed/refractory AML cases (4 of 8), with manageable CRS [113, 114]. In B‐cell malignancies, CD72 nanobody‐derived ‘H24’ CARs enhanced lysis through framework optimisation, informing resistance mechanisms observed in relapsed patients [115]. The immunoregulatory receptor LILRB1 functioned as a salvage target in CD19‐resistant B‐ALL/B‐NHL; CAR‐T cells eliminated CD19‐CAR–resistant clones and exhibited cross‐reactivity against monocytic AML [116].

### 3.3 Pan‐Cancer and Neoantigen Targets

Beyond lineage‐restricted antigens, metabolic dysregulation and tumour‐specific mutations broaden targeting options. Metabolic and stress‐induced antigens reveal shared vulnerabilities. SLC3A2, a cystine transporter markedly overexpressed in multiple tumours, increased CAR‐T efficacy in xenograft models with low toxicity [117] (Figure 6F). PS was tractable via two strategies: adaptor‐redirected CARs (EDA/BCMA CARs paired with annexin fusion proteins) demonstrated in vivo activity, and direct Anxa CAR‐T cells minimised fratricide by reducing CAR density and inhibiting kinases while maintaining antitumour efficacy [98].

Neoantigen‐directed approaches exploit tumour‐restricted mutations. High‐affinity scFvs recognising HLA‐A*11:01–restricted KRAS G12V improved NeoCAR activity in lung, pancreatic, and renal carcinomas; inducible IL‐12 co‐expression enhanced antigen presentation, and TCR knockout reduced alloreactivity [118] (Figure 6G). In spliceosome‐mutated AML/MDS, recurrent frameshift neoantigens including CLK3 and RHOT2 enabled selective destruction of mutant cells by TCR‐engineered T cells while sparing normal counterparts; however, endogenous T‐cell exhaustion necessitated exogenous TCR transfer [119] (Figure 6H). This set of targets spans tumour‐specific epitopes such as B7‐H3 in CNS tumours, haematopoietic checkpoints like CLL1 in AML, and pan‐cancer neoantigens including KRAS G12V, illustrating complementary strategies to counter immune escape. The expanding atlas of targetable antigens provides the specificity needed for effective and safe CAR‐T therapy, yet heterogeneity and antigen loss continue to demand combinatorial targeting and microenvironment‐responsive designs to achieve durable responses across malignancies.

### 3.4 Targeting Oncogenic Viral Antigens in Virus‐Associated Solid Tumours

Beyond the conventional self‐antigens or aberrantly expressed TAAs discussed in the preceding sections, a distinct and highly promising class of targets exists in virus‐associated malignancies. Oncogenic viruses are etiological agents in approximately 10%–20% of all human cancers worldwide, including cervical cancer (HPV), head and neck cancers (HPV), hepatocellular carcinoma (HBV, HCV), nasopharyngeal carcinoma, and certain lymphomas (EBV), as well as adult T‐cell leukaemia/lymphoma (HTLV‐1). These tumours represent a unique opportunity for CAR‐T cell therapy because they express oncogenic viral antigens that are entirely foreign to the host immune system, are causally linked to the malignant phenotype, and are often essential for tumour maintenance [120].

The conceptual advantages of targeting viral antigens over conventional TAAs are profound. First, the primary challenge in solid tumour CAR‐T therapy—on‐target/off‐tumour toxicity—is theoretically circumvented. Since viral proteins have no normal human homologue, their expression is strictly confined to tumour cells, minimising the risk of cross‐reactivity with healthy tissues. Second, the immunogenicity of foreign viral proteins can be superior to that of self‐antigens, potentially eliciting more robust and durable T‐cell responses. However, clinical experience with TAA‐specific engineered T cells has been associated with significant side effects, as these cells may react with and damage healthy tissue that expresses low levels of the target antigen [120]. Creating T cells that specifically target oncogenic viral antigens offers a direct strategy to avoid these cross‐reactive side‐effects.

Despite these theoretical advantages, the clinical translation of viral antigen‐specific CAR‐T cells presents a distinct set of challenges that must be addressed through integrated engineering. Viral mutation and integration site diversity constitute a primary hurdle. Oncogenic viruses, particularly those with error‐prone polymerases or those that integrate randomly into the host genome, can exhibit significant sequence heterogeneity. This can lead to the emergence of escape variants that no longer express the targeted epitope, mirroring the antigen loss problem seen with TAAs. For example, heterogeneity in HPV E6/E7 sequences or HBV surface antigen (HBsAg) variants could render a single‐target CAR ineffective. Antigen expression dynamics also pose a problem; latency‐associated viral antigens may be expressed at very low levels, limiting CAR recognition. Manufacturing complexity is another challenge, as it requires generating T cells that are both potent against the virus and able to persist in the immunosuppressive TME of chronic viral infections, where T‐cell exhaustion is often pre‐existing.

To overcome these obstacles, the principles of integrated engineering discussed in this review are directly applicable. Multi‐targeting strategies can be employed to prevent viral escape; for instance, a bispecific CAR targeting two conserved regions of the HPV E6 and E7 oncoproteins could mitigate the risk of mutational escape. Logic‐gated control could be designed to activate only in the presence of viral antigens, coupled with a safety switch to manage toxicity. Metabolic and epigenetic reprogramming are critical to overcome pre‐existing T‐cell exhaustion common in chronic viral diseases, while TME remodelling strategies are essential to break the long‐established immunosuppressive networks typical of virus‐driven tumours. The integration of viral antigen‐specific CARs with oncolytic viruses that express viral antigens or with checkpoint inhibitors represents another potent synergistic frontier. By applying the same rigorous engineering logic mapped out for conventional TAAs—precision, resilience, and TME modulation—the field can translate the unique specificity of viral antigens into durable clinical responses for a significant fraction of virus‐related cancers.

## 4 Synergistic Engineering for TME Remodelling and CAR‐T Resilience

Beyond target selection, achieving durable clinical efficacy in solid tumours requires CAR‐T cells that can actively counter and remodel the immunosuppressive TME. This section examines integrated co‐engineering approaches that couple cytokine circuit design with intrinsic metabolic and epigenetic reprogramming to disrupt local suppression, enhance cellular fitness, and engage endogenous immunity, enabling CAR‐T cells to function as environment‐responsive therapeutics. These strategies, which combine localised immunomodulation with intrinsic reprogramming to foster a sustained therapeutic milieu, are summarised in Table 3.

**TABLE 3:** Cytokine and metabolic engineering strategies.

CAR‐T type	Mechanism	Function	Cancer immunotherapy application	References
EGFRvIII CAR‐T and IL‐12	Local IL‐12 delivery promotes TME remodelling	Increased CAR‐T cytotoxicity; reduced tregs; activated myeloid cells	Glioblastoma (established tumours)	[126]
GD2 CAR‐T (IL‐15 armoured)	Retroviral IL‐15 transgenic expression	50% CR rate; enhanced brain penetration	Intracranial glioblastoma	[127]
CMC‐21 backpack CAR‐T	Nanoparticle‐enabled IL‐21 release and O_2_ generation/H^+^ neutralisation	Enhanced endogenous NK/T‐cell infiltration	Nutrient‐deprived hypoxic solid tumours	[29]
G6/7R CAR‐T	Chimeric receptor capturing IL‐6 and constitutive IL‐7 signalling	Depleted protumour IL‐6; enhanced proliferation/persistence	Hematologic and solid malignancies	[128]
GzB‐IL‐18 CAR‐T	Activation–dependent IL‐18 release via granzyme B cleavage	Enhanced cytotoxicity/proliferation; avoided systemic toxicity	αβ/γδ CAR‐T applications	[129]
Zip18R C.TNC CAR‐T	Leucine zipper‐stabilised IL‐18 receptor	Enhanced cytokine secretion and tumour rechallenge protection	Paediatric DIPG, osteosarcoma, rhabdomyosarcoma	[130]
BC2 CAR‐T (CXCR2and)	CXCR2‐mediated trafficking along radiotherapy‐induced IL‐8 gradients	Improved metabolic fitness and metastatic clearance	Paediatric sarcomas	[131]
7XCL1‐CAR‐T	CEA‐targeted CAR secreting XCL1/IL‐7 upon engagement	cDC1 recruitment; endogenous neoantigen‐specific CD8^+^ T‐cell priming	Heterogeneous colorectal cancer	[132]
Fla‐expressing CAR‐T	Bacterial flagellin‐induced macrophage/DC activation	‘Cold’ to ‘hot’ TME conversion; broadened antigen coverage	Antigen‐heterogeneous solid tumours	[133]
Host‐dependent IL‐23 CAR‐T	Host‐derived IL‐23 supports CAR‐T function	Sustained expansion/persistence; inflammatory TME conditioning	Cancers requiring host immune engagement	[134]
Exosome‐mimetic CAR	MHC–peptide/CD86 presentation and anti‐CD3/EGFR bispecifics	Endogenous T‐cell activation; PD‐L1 blockade synergy	Broad solid tumours	[135]
TENG electro‐immuno CAR‐T	Friction‐induced DC current for immunogenic cell death	60% tumour mass reduction; M1‐like macrophage polarisation	Immunosuppressive solid tumours	[136]
PPZ‐potentiated MSLN CAR‐T	Proton pump inhibition normalises lysosomal pH	Restored MSLN membrane expression	Breast/NSCLC brain metastases	[137]
MCT‐1 blocked CAR‐T	Pharmacological inhibition of lactate export	Disrupted tumour lactate shuttling; enhanced cytotoxicity	B‐cell lymphoma	[138]
BCKDK‐overexpressing CAR‐T	Enhanced branched‐chain amino acid (BCAA) catabolism	Prolonged survival; reduced exhaustion	Metabolic stress‐resistant tumours	[139]
DCA‐reprogrammed CAR‐T	Pyruvate dehydrogenase kinase inhibition leads to OXPHOS shift	Improved metabolic flexibility and stemness	Glucose‐dependent malignancies	[58]
OXPHOS‐enhanced CAR‐T	Pharmacologic/genetic promotion of oxidative phosphorylation	Central memory phenotype; reduced exhaustion during chronic antigen	Recurrent antigen exposure scenarios	[140]
NAD^+^‐restored geriatric CAR‐T	CD38 inhibition and NAD^+^ precursors	Reversed mitochondrial dysfunction and stemness loss	Elderly patients with T‐cell senescence	[48]
P4HA1‐targeted T cells	Reversal of P4HA1‐mediated TCA cycle disruption	Enhanced precursor CD8^+^ T‐cell expansion	Solid tumours with dysfunctional TILs	[59]
Vitamin C‐primed CAR‐T	TET2‐mediated DNA demethylation	Suppressed epigenetic exhaustion; enhanced expansion/cytokine production	Tumours requiring durable T‐cell persistence	[141]
Butyrate‐treated CAR‐T	HDAC inhibition via microbiome metabolite	Central memory‐like phenotype through histone modification	Cancers needing enhanced T‐cell persistence	[142]
Glutamine‐restricted CAR‐T	mTORC1/epigenetic modulation of differentiation	Effector memory‐like phenotype with improved persistence	Solid tumours requiring long‐term surveillance	[143]
HDACi‐modulated CAR‐T	Entinostat‐mediated epigenetic reprogramming	Enhanced mitochondrial metabolism and anti‐apoptotic capacity	Repeated antigen challenge settings	[75]

### 4.1 Cytokine Circuit Engineering

**FIGURE 7:**
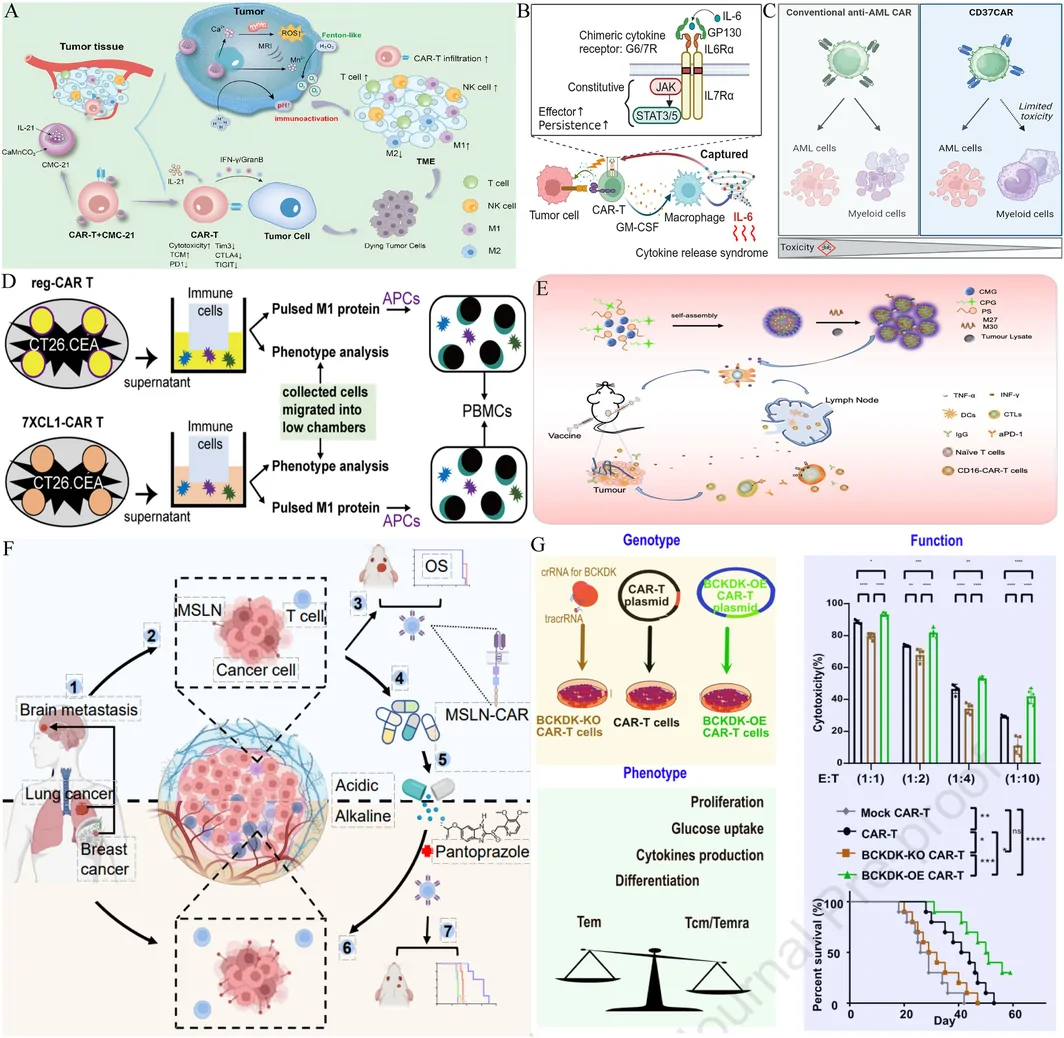
Advanced engineering and combinatorial strategies for optimizing CAR‐T cell efficacy through metabolic rewiring, TME remodelling, and synergistic immunotherapy. Schematic illustration of the ‘Vitality Backpack’ strategy, wherein CAR‐T cells functionalized with CaMnCO_3_/IL‐21 nanoparticles (CMC‐21) nanoparticles synergistically remodel the tumour microenvironment to sustain effector function and potentiate solid tumour eradication. Reproduced with permission [29]. Copyright from John Wiley and Sons, 2025. (B) Schematic illustration: Development of a chimeric cytokine receptor that captures IL‐6 and enhances the antitumor response of CAR‐T cells. Reproduced with permission [128]. Copyright from Elsevier Ltd., 2024. (C) Schematic illustration: CD37 is a safe chimeric antigen receptor target to treat acute myeloid leukaemia. Reproduced with permission [129]. Copyright from Elsevier Ltd., 2024. (D) Schematic of the ex vivo recruitment and functional verification assay. Conditioned media from CT26. CEA tumour cells co‐cultured with reg‐CAR T or 7XCL1‐CAR T cells were employed as chemoattractants in a transwell system. Migrated immune cells were harvested for phenotypic analysis and functionally validated via an M1 protein pulsing assay to confirm their capacity to activate PBMCs as antigen‐presenting cells. Reproduced with permission [132]. Copyright from BMJ Publishing Group Ltd., 2025. (E) Schematic illustration of the assembly and synergistic antitumor mechanism of a combinatorial immunotherapy strategy integrating nanoparticle‐based vaccination, CD16‐CAR‐T cells, and anti‐PD‐1 blockade. CpG ODN is encapsulated via self‐assembly with CMG and PS to form nanoparticles (CNPs), which are subsequently formulated with tumour‐derived antigens to construct the therapeutic vaccine. Upon administration, this regimen elicits a robust immune response characterized by the generation of antigen‐specific CTLs and tumour‐specific antibodies; crucially, these antibodies direct CD16‐CAR‐T cells to specifically target and eliminate tumour cells via antibody‐dependent cellular cytotoxicity (ADCC), achieving enhanced therapeutic efficacy when combined with immune checkpoint inhibition. Reproduced with permission [135]. Copyright from Springer Nature, 2023. (F) Schematic illustration: Proton pump inhibitor attenutes acidic microenvironment to improve the therapeutic effects of MSLN‐CAR‐T cells on the brain metastasis of solid tumours. Reproduced with permission [137]. Copyright from Elsevier Ltd., 2025. (G) Schematic illustration: Branched‐chain ketoacid dehydrogenase kinase (BCKDK) modification enhances the anticancer efficacy of CAR‐T cells by reprogramming branched chain amino acid (BCAA) Metabolism. Reproduced with permission [139]. Copyright from Elsevier Ltd., 2024.

Cytokine circuit engineering—which involves genetically programming CAR‐T cells to produce or respond to cytokines in a spatially and temporally controlled manner—has emerged as a core strategy for counteracting the immunosuppressive TME. The overarching strategy is to provide localised immunostimulation while minimising systemic toxicity, which can be achieved through several complementary approaches. Localised cytokine delivery directly to the tumour site—such as intratumoural administration of IL‐12 or the use of nanoparticle ‘fitness backpacks’ (e.g., CaMnCO_3_/IL‐21) that neutralise acidity and release IL‐21—has shown promising efficacy in enhancing CAR‐T and endogenous immune cell function in preclinical models [126]. GD2 CAR‐T cells co‐expressing IL‐15 improved intracranial control in aggressive glioblastoma models, with a 50% complete response rate relative to conventional constructs [127]. Calcium‐manganese carbonate nanoparticles co‐loaded with IL‐21 (CaMnCO_3_/IL‐21, CMC‐21) functioned as ‘fitness backpacks,’ neutralising acidity, generating oxygen from H_2_O_2_, and releasing IL‐21 to increase infiltration and cytotoxicity of CAR‐T and endogenous immune cells [29] (Figure 7A).

Autonomous, engineered cytokine circuits within CAR‐T cells offer a more integrated solution. These include chimeric receptors like G6/7R that convert extracellular IL‐6 into constitutive IL‐7 signalling, and conditionally activated cytokine precursors that release payloads specifically upon CAR‐T cell activation, thereby coupling cytokine activity to tumour recognition [128, 129] (Figure 7B,C). Paediatric tumour–targeted CAR‐T cells expressing Zip18R (leucine zipper–stabilised IL‐18R) showed increased cytokine production, proliferation, and in vivo activity [130].

To improve directional trafficking and immune contexture remodelling, CAR‐T cells can be engineered to express chemokine receptors to follow radiotherapy‐induced gradients (e.g., IL‐8), or to secrete chemokines like XCL1 along with cytokines like IL‐7 to recruit and prime antigen‐presenting cells, fostering a supportive immune niche [131, 132] (Figure 7D).

Activating endogenous immunity expanded control of heterogeneous disease. CAR‐T cells engineered to express flagellin C (FlaC) secreted bacterial flagellin, activating macrophages and dendritic cells to remodel immunologically ‘cold’ TMEs and augment endogenous tumour‐specific CD8^+^ T cell responses [133]. In the absence of host‐derived IL‐23, CAR‐T growth, persistence, and inflammatory conditioning were substantially compromised [134]. Antigen‐fed dendritic cells produced antibody‐engineered exosomes that transferred MHC–peptide/CD86 complexes to endogenous T cells; bispecific anti‐CD3/anti‐EGFR antibodies enhanced T‐cell/tumour engagement, and anti‐PD‐L1 mitigated compensatory evasion [135] (Figure 7E). Friction‐based triboelectric nanogenerator (TENG) electro‐immunotherapy applied pulsed DC current to kill immunogenic cells, mature dendritic cells and counter suppression; M1‐like macrophage polarisation and Treg reduction increased infiltration and tumour clearance with CAR‐T treatment, reducing tumour mass by 60% [136]. These diverse approaches share the common goal of reshaping the TME from immunosuppressive to immunostimulatory. However, key challenges include ensuring precise spatial control to avoid systemic toxicity, achieving durable circuit function in vivo, and preventing the induction of compensatory immunosuppressive pathways or excessive inflammation that could lead to toxicity or T‐cell exhaustion. The optimal cytokine combination, regulation logic, and delivery method are likely tumour‐context dependent.

### 4.2 Metabolism‐Immune Co‐Reg

Cytokine support is frequently insufficient without intrinsic metabolic interventions to sustain CAR‐T fitness in nutrient‐deprived, acidic, and hypoxic TMEs. Microenvironment‐directed measures can directly augment antigen display. In breast cancer brain metastases, acidic TMEs reduced membrane MSLN; pantoprazole (PPZ) elevated intratumoural pH, blocked lysosomal MSLN degradation, and markedly enhanced CAR‐T cytotoxicity [137] (Figure 7F).

To bolster resilience, metabolic pathways within CAR‐T cells have been directly engineered. Activated CAR‐T cells increased lactate export via MCT‐1/MCT‐4; CAR‐T cells tolerated MCT‐1 inhibition, but tumour MCT‐1 blockade disrupted lactate shuttling and improved lymphoma clearance in vivo [138]. Branched‐chain amino acid (BCAA) metabolism influenced potency: BCAA supplementation increased cytotoxicity, and branched‐chain ketoacid dehydrogenase kinase (BCKDK) overexpression enhanced efficacy and survival in experimental models [139] (Figure 7G). DCA shifted hyper‐glycolytic, in vitro–expanded T cells toward OXPHOS, improving metabolic flexibility, stemness, and in vivo efficacy [58].

Optimising mitochondrial fitness supported endurance. Pharmacologic and genetic enhancement of OXPHOS relative to glycolysis promoted central memory–like phenotypes, increasing persistence and reducing exhaustion under chronic antigen exposure [140]. Age‐related NAD^+^ depletion driven by CD38 upregulation impaired mitochondrial function; restoring NAD^+^ via CD38 inhibition or precursors improved the performance of geriatric CAR‐T cells [48]. The mitochondrial enzyme P4HA1 accumulated in exhausted CD8^+^ T cells, disrupted the TCA cycle, and promoted dysfunction; targeting P4HA1 expanded precursor CD8^+^ T cells and augmented systemic immunity [59].

Epigenetic priming further improved resilience. Vitamin C (ascorbate) facilitated TET2‐mediated DNA demethylation during manufacturing, inhibiting exhaustion programs and promoting memory phenotypes with enhanced in vivo expansion [141]. Butyrate, a microbiome‐derived short‐chain fatty acid, reinforced central memory–like features via histone modification [142]. Glutamine restriction skewed differentiation toward effector memory phenotypes through mTORC1 and epigenetic control [143]. HDAC inhibitors such as entinostat enhanced memory differentiation, mitochondrial metabolism, and persistence after antigen rechallenge [75]. Combined cytokine‐armoured CAR‐T platforms and metabolically tuned designs—for example, IL‐12 co‐delivery in glioblastoma and DCA‐driven OXPHOS conversion—illustrated the utility of co‐engineering in suppressive TMEs.

Cytokine autonomy paired with metabolic resilience enables CAR‐T cells to maintain activity and adapt within the TME. Systematic integration of these modalities is required to translate engineered cellular functions into durable responses in solid tumours.

Bridging preclinical promise to clinical reality in cellular fitness engineering. While compelling preclinical data support metabolic and epigenetic reprogramming, their translation into robust clinical benefit remains to be fully established. Strategies are moving beyond in vitro preconditioning to direct genetic engineering. A first‐in‐human phase I trial (NCT05715606) is evaluating GD2‐targeted CAR‐T cells genetically modified to overexpress the glucose transporter GLUT1 for neuroblastoma, directly testing the hypothesis that enhancing glycolytic capacity can improve persistence. Early‐phase clinical trials have also begun to explore the combination of CAR‐T therapy with pharmacologic modulators. For instance, the combination of mesothelin‐targeted CAR‐T cells with the histone deacetylase inhibitor sodium valproate (VPA) in an ovarian cancer trial showed enhanced CAR‐T cytotoxicity and improved tumour infiltration, providing initial clinical evidence for epigenetic pharmacologic modulation [144]. However, critical translational questions persist. The metabolic landscape of human solid tumours is vastly more complex and heterogeneous than in murine models. An intervention that boosts OXPHOS may be ineffective in a profoundly hypoxic niche. Furthermore, engineering for a persistent, memory‐like state might inadvertently blunt the immediate cytotoxic capacity needed for tumour clearance—a delicate balance between ‘fitness’ and ‘potency’ that is difficult to predict from mouse studies. The long‐term genetic and functional stability of such engineered cells in patients is unknown. Therefore, while metabolic and epigenetic reprogramming are rational strategies to counter TME‐driven exhaustion, their clinical translation necessitates a deeper understanding of human tumour biology and the development of reliable biomarkers to identify patients most likely to benefit.

A critical appraisal of metabolic reprogramming reveals several unresolved challenges. First, the metabolic landscape of the human TME is vastly more complex and variable than in murine models. Interventions like shifting T cells toward OXPHOS may not be universally beneficial across all tumour types, as some niches might be profoundly hypoxic or lack critical nutrients, rendering OXPHOS inefficient. Second, there is a potential conflict between optimising T‐cell fitness and maintaining antitumour effector functions. Highly glycolytic metabolism is often associated with robust effector differentiation and cytokine production. Over‐engineering for a persistent, memory‐like metabolic state might inadvertently blunt the immediate cytotoxic capacity needed for tumour clearance. Third, the long‐term genetic and functional stability of metabolically engineered CAR‐T cells in patients is unknown. Unintended consequences on epigenetic programming or the risk of fostering oxidative stress and DNA damage require careful long‐term monitoring. Finally, many metabolic interventions are administered systemically, which may have off‐target effects on normal tissues. The development of T‐cell‐intrinsic, self‐regulated metabolic circuits that dynamically adapt to local nutrient availability represents a promising but highly complex future direction.

## 5 Remodelling the Immunosuppressive and Physical TME

Beyond intrinsic T‐cell engineering, clinical efficacy in solid tumours depends on dismantling immunosuppressive and physical barriers within the TME. This section addresses strategies that deplete suppressive cellular networks and circumvent anatomical constraints to establish conditions permissive for CAR‐T‐cell activity.

### 5.1 Targeting Immunosuppressive Cellular Networks

Immunosuppressive myeloid and lymphoid populations residing within the TME actively attenuate CAR‐T‐cell function through multiple mechanisms. TAMs, especially the M2‐polarised subsets, suppress CAR‐T cell cytotoxicity, activation, and cytokine production. This suppression is often associated with upregulated PD‐L1 expression following CAR‐T engagement. PD‐L1 blockade, but not PD‐1 inhibition, restored activity primarily by depleting M2 macrophages rather than solely through checkpoint reversal, supporting PD‐L1 inhibitors with CAR‐T therapy in macrophage‐rich tumours [145]. Depletion of specific immunosuppressive TAM subsets, such as those expressing MARCO, represents another direct strategy [146]. Mitigating the function of regulatory T cells (Tregs) through concurrent depletion during therapy has shown benefit in preclinical models [147].

Direct disruption of suppressive signalling through genetically engineered CAR‐T cells has significantly strengthened antitumour activity. SIRPα‐Fc‐secreting (Sirf) CAR‐T cells—which secrete the CD47 blocker SIRPα‐Fc—outperformed conventional CAR‐T cells in solid tumours by neutralising ‘don't eat me’ signals, promoting a central memory phenotype, increasing intratumoural persistence, reducing myeloid‐derived suppressor cells (MDSCs), and expanding stimulatory dendritic cells and M1 macrophages [53]. CD19‐s47‐CAR‐T cells secreting an anti‐CD47 scFv improved degranulation, cytokine production, persistence, and multifunctionality; localised CD47 blockade modulated macrophage phagocytosis and polarisation while potentially reducing systemic toxicity associated with anti‐CD47 antibodies [54].

**FIGURE 8:**
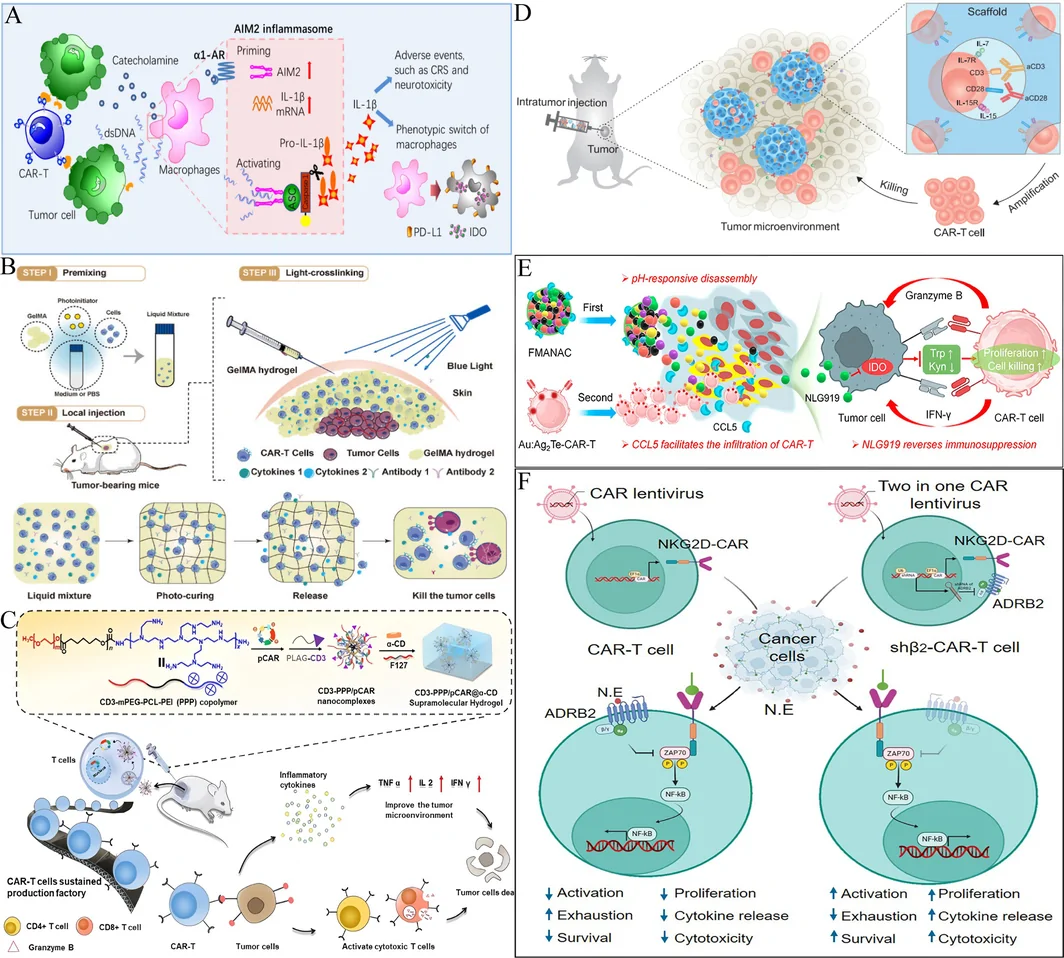
Advanced bioengineering strategies for in situ programming, localized delivery, and signalling modulation to optimize CAR‐T cell therapy. (A) Schematic diagram of the absent in melanoma 2 (AIM2) inflammasome and α_1_‐AR signalling pathway. This schematic diagram illustrates the critical roles of the AIM2 inflammasome and the α_1_‐adrenergic receptor (α_1_‐AR) in facilitating IL‐1β release and macrophage‐mediated immunosuppression triggered by CAR‐T treatment. In the context of cytokine release syndrome (CRS), the figure highlights the involvement of key molecular components, including double‐stranded DNA (dsDNA) signalling, as well as the upregulation of immunosuppressive markers such as indoleamine 2,3‐dioxygenase (IDO) and programmed cell death‐ligand 1 (PD‐L1). Reproduced with permission [150]. Copyright from BMJ Publishing Group Ltd., 2021. (B) Schematic illustration of the implantation procedure for the injectable delivery system. Initially, CAR‐T cells and cytokines are encapsulated within the hydrogel precursor solution. Following this, the mixture is administered via injection directly into the tumour site. Subsequent exposure to blue light irradiation induces photo‐cross‐linking, leading to in situ gelation. As a result, the hydrogel forms a locally solidified matrix that ensures sustained release of CAR‐T cells, enabling continuous targeting and elimination of malignant cells. Reproduced with permission [151]. Copyright from Elsevier Ltd., 2022. (C) Schematic diagram illustrating the in situ programming of CAR‐T cells within the tumour microenvironment by exploiting the infiltrative characteristics of a gene‐loaded supramolecular hydrogel system for solid tumour therapy. Reproduced with permission [152]. Copyright from John Wiley and Sons, 2024. (D) Schematic diagrams depict the artificial lymph node‐like scaffold constructed from aCD3 and aCD28 antibody‐functionalized porous microspheres that supply stimulatory and co‐stimulatory signals to support T cell expansion, alongside the in situ programming of CAR‐T cells within the tumour microenvironment by leveraging the infiltrative characteristics of a gene‐loaded supramolecular hydrogel system for solid tumour treatment. Reproduced with permission [153]. Copyright from Oxford University Press, 2024. (E) Schematic illustration: Tumour microenvironment‐responsive nano‐immunomodulators for enhancing chimeric antigen receptor‐T cell therapy in lung cancer. Reproduced with permission [154]. Copyright from American Chemical Society, 2025. (F) Schematic illustration: Intrinsic beta‐2 adrenergic receptor (ADRB2) inhibition improves CAR‐T cell therapy efficacy against prostate cancer. Reproduced with permission [155]. Copyright from Elsevier Ltd., 2024.

Targeting alternative immune checkpoints beyond the PD‐1/PD‐L1 axis, such as VISTA, is also being explored to augment endogenous immunity alongside CAR‐T activity [148]. More sophisticated engineering allows CAR‐T cells to convert inhibitory signals into stimulatory ones; for example, bispecific CAR‐T cells targeting IL‐13Rα2 and TGF‐β can sequester and functionally invert TGF‐β within the glioblastoma TME [149]. Beyond receptor‐based modifications, intrinsic polarisation of CAR‐T cells during manufacturing can yield products with superior fitness. Th9‐polarised CAR‐T cells exhibit a central memory phenotype, reduced exhaustion, and enhanced persistence and tumour control compared to conventional Th1‐polarised cells, demonstrating the impact of pre‐infusion cellular state [150] (Figure 8A). Collectively, these strategies aim to engineer CAR‐T cells that are not merely resistant to suppression but are active participants in remodelling the immunosuppressive landscape. The translational challenge lies in achieving a sufficient local effect without triggering systemic autoimmunity or excessive inflammation, and in identifying the dominant immunosuppressive mechanisms in specific tumour types to guide rational combination.

Physical barriers necessitated tailored delivery and modulation. Biomaterial platforms created local reservoirs and sustained release. Injectable, photocurable gelatin methacrylamide (GelMA) hydrogels supported CAR‐T survival and proliferation within the TME, prolonged retention, and enabled gradual release, enhancing efficacy and survival relative to local or intravenous administration in melanoma models [151] (Figure 8B). More advanced systems facilitate in situ CAR‐T generation or act as protected ‘incubators’ [152, 156] (Figure 8C).

Biomimetic scaffolds provided structured support. Lymph node–inspired Poly(lactic‐co‐glycolic acid) (PLGA) scaffolds incorporating anti‐CD3/CD28 and cytokines functioned as integrated platforms for CAR‐T loading, delivery, activation, and expansion, supporting ~50‐fold proliferation while maintaining cytotoxicity and enabling sustained activation and expansion within tumours for up to 30 days, delaying progression [153] (Figure 8D). Localised intracavitary administration has demonstrated clinical feasibility and improved therapeutic index by achieving high local concentrations and minimising systemic exposure, validating the principle of circumventing circulatory barriers [157, 158].

TME‐responsive nanotechnologies adapted local conditions to facilitate CAR‐T activity. The acid‐sensitive nanomodulator, a TME‐responsive nanoimmunomodulator (FMANAC), dissociated in acidic TME, releasing CCL5 to enhance chemokine gradients for CAR‐T recruitment and NLG919 to counter IDO‐mediated suppression; with NIR‐II imaging guidance and CD276 CAR‐T cells, FMANAC improved tumour control in lung cancer by remodelling the TME [154] (Figure 8E). Addressing antigen heterogeneity and scarcity, the Myc‐pHLIP peptide used acidic pH to anchor a surrogate c‐Myc tag on tumour cell surfaces; pre‐treatment enabled recognition and killing by Myc‐targeted CAR‐T cells or anti‐Myc antibodies, providing a generalisable route to redirect immune therapies against antigen‐diverse solid tumours [159].

Combining CAR‐T cells with oncolytic adenoviruses (OAds) augmented activity. CD70 CAR‐T cells engineered to deliver third‐generation OAds (e.g., TS‐2021) enhanced control of glioblastoma; TS‐2021–infected CAR‐T cells (CAR‐TTS‐2021) secreted IL‐15 via an autocrine mechanism, reduced exhaustion markers, and maintained function under persistent antigen exposure, countering antigen heterogeneity and microenvironmental resistance [160]. Tumour cells also upregulated resistance mechanisms such as the complement regulator CD46 following CAR‐T engagement; CD46 inhibited granzyme B within the immunologic synapse. Blocking CD46 restored cytotoxicity and identified a tractable target to improve efficacy [161].

### 5.2 Disrupting Physical and Stromal Barriers

Beyond cellular immunosuppression, dense stromal matrices and anatomical confinement necessitate strategies that modulate physical barriers. Direct engagement of tumour stroma addresses core obstacles to infiltration. Cancer‐associated fibroblasts (CAFs) expressing fibroblast activation protein (FAP) impede T‐cell trafficking and diminish CAR‐T cytotoxicity. ‘Smart CAR‐T cells’ engineered via transcription activator‐like effector nuclease (TALEN)‐mediated gene editing co‐expressed a constitutive CAR targeting FAP and an inducible CAR recognising a tumour‐associated antigen (TAA), which improved infiltration and cytotoxicity while reducing off‐tumour risk [155] (Figure 8F). Physiologically relevant screening platforms further inform combination design. A 3D‐bioprinted vascularised breast tumour model, incorporating heterogeneous tumour spheroids and perfused vasculature, enabled real‐time assessment of angiogenesis, invasion, and treatment response; HER2‐directed CAR‐T cells achieved marked activation, infiltration, and approximately 70% tumour volume reduction, with synergy observed for vascular‐targeted strategies [162].

Integrated approaches can simultaneously counter immunosuppression and stromal resistance. An IL‐13Rα2/TGF‐β bispecific CAR‐T platform converted TGF‐β into an activating cue specifically within the glioblastoma TME, significantly increasing CAR‐T infiltration and reducing immunosuppressive myeloid populations, outperforming standard IL‐13Rα2 CAR‐T cells in patient‐derived xenografts and syngeneic models [149]. Enhanced stromal targeting was achieved with ‘smart’ allogeneic CAR‐T cells engineered by TALEN to co‐express a constitutive FAP‐directed CAR and an inducible TAA‐directed CAR, improving infiltration and cytotoxicity in stroma‐dense solid tumours while mitigating off‐tumour toxicity from continuous FAP engagement [155]. Tumour adaptation also involves upregulation of complement regulators. CD46 increased after CAR‐T engagement and attenuated cytotoxicity by suppressing granzyme B activity at the immunologic synapse; antibody‐mediated CD46 blockade restored CAR‐T killing, nominating CD46 as a co‐target to counter this evasion mechanism [161].

Precise spatiotemporal control over CAR‐T cell localisation and activation is critical. For example, the TME‐responsive nanomodulator FMANAC exploits tumour acidity to release CCL5 (enhancing CAR‐T chemotaxis) and NLG919 (countering IDO‐mediated suppression). When guided by NIR‐II imaging and combined with CD276‐targeted CAR‐T cells, FMANAC improved antitumour efficacy in lung cancer models by remodelling the local milieu [154]. The Myc‐pHLIP peptide offered a versatile tagging solution for variable or low‐abundance antigens by anchoring a surrogate c‐Myc tag to tumour and stromal cell surfaces in a pH‐dependent manner; subsequent Myc‐targeted CAR‐T cells or antibodies achieved effective recognition and killing, addressing antigen scarcity in solid tumour immunotherapy [159]. Oncolytic virotherapy provided complementary modulation. CD70‐CAR‐T cells delivering third‐generation oncolytic adenoviruses (TS‐2021) produced IL‐15 via autocrine signalling; CAR‐TTS‐2021 reduced exhaustion markers and maintained function under chronic antigen exposure, improving control in glioblastoma despite antigen heterogeneity and suppressive TME pressures [160].

Biomaterial platforms offer a powerful means to support sustained, localised CAR‐T activity while simultaneously limiting systemic exposure. Injectable GelMA hydrogels function as supportive matrices that improve the retention and survival of CAR‐T cells within the TME, thereby enhancing antitumour efficacy and overall survival compared with bolus local or intravenous administration in melanoma models [151]. TSPs composed of biodegradable GelMA/PEGDA functioned as in vivo incubators; peritumoural delivery of mesothelin‐targeted CAR‐T cells via TSPs exceeded systemic or intratumoural injection by providing protected expansion and controlled release [156]. Injectable supramolecular hydrogels formed through α‐cyclodextrin/mPEG‐PCL‐PEI host–guest interactions and coated with anti‐CD3–polyglutamate enabled in situ CAR‐T manufacture by delivering plasmid DNA encoding a CAR under a T‐cell–specific promoter (pCAR), driving localised activation, proinflammatory cytokine production, and cytotoxic infiltration [152]. Lymph node–mimetic PLGA scaffolds incorporating anti‐CD3/CD28 and cytokines supported loading, activation, and prolonged expansion, achieving approximately 50‐fold proliferation while maintaining cytotoxicity and sustaining function in tumours for up to 30 days, thereby delaying progression [153].

Innovative physical modulation strategies are being combined with CAR‐T therapy to overcome stromal barriers and enhance infiltration. Selective irreversible electroporation (sIRE), tuned to lyse cancer cells while sparing immune cells, was shown to generate a chemokine gradient that promotes the migration and tumour infiltration of systemically administered CAR‐T cells. Repeated sIRE applications combined with CAR‐T cells demonstrated synergistic therapeutic benefit in models of malignant pleural mesothelioma, offering a clinically feasible method to condition the TME [163]. Furthermore, nanotechnology is providing multi‐faceted solutions to enhance CAR‐T therapy. Nanoparticle‐based strategies can optimise genetic modification, enhance tumour site infiltration, modulate the immunosuppressive TME, mitigate antigen heterogeneity, and enable real‐time monitoring [164]. For example, microfluidic technologies are being leveraged to create controlled microenvironments for cell delivery, improving early retention, engraftment, and providing immunoisolating architectures that could be highly beneficial for localised CAR‐T therapy in solid tumours [165]. Beyond these examples, nanoparticle (NP) platforms are emerging as versatile and powerful tools that synergise with CAR‐T cell engineering to overcome the multi‐layered barriers of solid tumours [166]. NPs offer several distinct advantages that align perfectly with the principles of integrated engineering. First, in manufacturing and delivery, NPs can be designed for non‐viral, efficient, and safer CAR gene delivery, potentially reducing the cost and complexity of ex vivo manufacturing. Second, to enhance trafficking and infiltration, NPs functionalised with targeting ligands or responsive to TME stimuli (e.g., pH, enzymes) can actively guide CAR‐T cells to the tumour site or disrupt the dense extracellular matrix, overcoming physical barriers. Third, for TME remodelling, NPs can serve as localised depots for immunomodulatory payloads like cytokines or checkpoint inhibitors, converting an immunosuppressive ‘cold’ TME into a ‘hot’ one without the systemic toxicities associated with systemic administration. Fourth, to tackle antigen heterogeneity, NPs can be used for in situ tumour ‘painting’ by coating tumour cells with synthetic antigens, making them recognisable to universal CAR‐T cells. Finally, for real‐time monitoring, NPs can be integrated as trackable imaging probes to non‐invasively monitor CAR‐T cell biodistribution and activity, enabling precise management of therapeutic efficacy and toxicity. The synergy between NPs and AI further amplifies this potential; AI algorithms can analyse NP properties and biological data to predict the optimal NP design for a given CAR‐T construct and tumour type, accelerating the translation from bench to bedside. This convergence positions NPs not merely as delivery vehicles but as integral components of the next‐generation ‘adaptive therapeutic system’.

Targeted delivery to confined anatomical spaces improved local control and reduced systemic toxicity. Intraperitoneal administration via hydrogels created a CAR‐T reservoir that better controlled peritoneal carcinomatosis from ovarian and pancreatic cancers than intravenous infusion, with lower systemic cytokine release [157]. Intrapleural delivery of mesothelin‐targeted CAR‐T cells produced higher tumour‐localised concentrations and superior efficacy with reduced systemic toxicity compared with intravenous administration in MPM models [158]. In vitro platforms remain important for optimising combinatorial strategies. A 3D‐bioprinted vascularised breast tumour system facilitated measurement of real‐time responses; HER2‐directed CAR‐T cells achieved notable activation and substantial infiltration with up to 70% tumour reduction, supporting its utility for predicting in vivo effects and guiding combinations [162]. Table 4 provides an overview of these approaches and their immunologic implications.

**TABLE 4:** Strategies for TME remodelling and enhanced CAR‐T delivery.

CAR‐T type/platform	Mechanism	Function	Cancer immunotherapy application	References
PD‐L1‐blocking CAR‐T	M2 TAM depletion via PD‐L1 blockade (not PD‐1)	Reversal of TAM‐mediated CAR‐T suppression	Macrophage‐rich solid tumours	[145]
MARCO‐KO CAR‐T	Resistance to TAM‐mediated phagocytosis	Enhanced persistence and tumour control	Solid tumours with MARCOand TAM infiltration	[146]
Treg‐depleted CAR‐T	Concurrent Treg depletion during administration	IL‐2 conservation; checkpoint molecule neutralisation	Immunosuppressive solid tumours	[147]
Sirf CAR‐T	SIRPα‐Fc secretion blocking CD47 ‘don't eat me’ signal	Reduced MDSCs; increased M1 macrophages and DCs; TME reprogramming	FAPand solid tumours	[53]
CD19‐s47 CAR‐T	Localised anti‐CD47 scFv delivery	Enhanced degranulation and cytokine production; reduced systemic toxicity	CD19and malignancies	[54]
VISTA‐blocking CAR‐T	VISTA checkpoint inhibition in IFN‐γ‐deficient TME	Enhanced endogenous T‐cell proliferation/cytotoxicity	B‐ALL/lymphoma/melanoma	[148]
IL‐13Rα2/TGF‐β bsCAR‐T	TGF‐β conversion from immunosuppressor to T‐cell activator	Increased T‐cell infiltration; myeloid suppression	Glioblastoma (patient‐derived xenografts)	[149]
Th9‐polarised CAR‐T	IL‐4/TGF‐β‐driven polarisation; high IL‐9/low IFN‐γ secretion	Enhanced tumour migration; long‐lived effector memory transition	Established hematologic/solid tumours	[150]
GelMA hydrogel CAR‐T	Photocurable hydrogel for sustained CAR‐T release	Prolonged intratumoural retention (> 7 days)	Melanoma (local/regional delivery)	[151]
TSP CAR‐T delivery	GelMA/PEGDA toroidal spirals as in vivo cell incubators	High‐capacity cell loading; controlled degradation	Mesothelinand solid tumours (peritumoural delivery)	[156]
In situ pCAR hydrogel	Host‐guest supramolecular hydrogel with anti‐CD3‐PGA coating	Tumour‐localised CAR‐T manufacturing	Solid tumours (T‐cell specific CD2 promoter)	[152]
LN‐Mimetic PLGA Scaffold	Microfluidic PLGA scaffold with anti‐CD3/CD28/cytokines	50‐fold CAR‐T expansion; 30‐day intratumoural persistence	Solid tumours (sustained activation)	[153]
i.p. Hydrogel CAR‐T	Injectable hydrogel scaffold for locoregional delivery	Minimised systemic exposure; enhanced local efficacy	Ovarian/pancreatic peritoneal carcinomatosis	[157]
Intrapleural CAR‐T	Localised delivery to pleural cavity	10‐fold higher tumour concentration vs. intravenous	Malignant pleural mesothelioma	[158]
FMANAC nanomodulator and CAR‐T	Acidic TME‐triggered CCL5/NLG919 release	Improved CAR‐T chemotaxis; IDO‐mediated immunosuppression reversal	Lung cancer (NIR‐II guided)	[154]
Myc‐pHLIP priming	pH‐dependent c‐Myc tagging of tumour/stroma	Universal targeting of antigenically diverse tumours	Solid tumours lacking suitable antigens	[159]
CAR‐TTS‐2021	OAd delivery and IL‐15 autocrine secretion	Reduced exhaustion under chronic antigen exposure	Glioblastoma (overcoming heterogeneity)	[160]
Anti‐CD46 CAR‐T	CD46 blockade restoring granzyme B activity	Overcoming adaptive tumour resistance	Cancers with post‐engagement CD46 upregulation	[161]
Smart Dual‐CAR‐T	TALEN‐edited constitutive FAP‐CAR and inducible TAA‐CAR	Stromal disruption; reduced off‐tumour toxicity	CAF‐rich solid tumours	[155]
3D‐bioprinted tumour model	Vascularised tumour spheres for therapy screening	70% tumour reduction; CAR‐T infiltration validation	Breast cancer (personalised therapy platform)	[162]

Progress has been driven by concurrent modulation of multiple TME components. CAF depletion with FAP‐CAR‐T cells paired with TAA targeting, blockade of the CD47/SIRPα axis using engineered Sirf or s47‐secreting CAR‐T cells, and VISTA inhibition to augment endogenous immunity constitute complementary measures against immunosuppression [53, 54, 148, 155]. Localised delivery enabled by biomaterials and TME‐responsive nanomaterials addresses physical and chemical constraints [151, 154, 156, 157, 158, 159]. Th9 polarisation improves intrinsic persistence and migratory capacity [150]. Incorporating oncolytic adenoviruses helps counter local immune evasion and antigen loss [160]. Effective CAR‐T therapy in solid tumours requires simultaneous depletion of suppressive cell populations such as TAMs and Tregs and deployment of biomaterials and nanotechnology to breach stromal and anatomical defences, thereby converting the TME into a setting permissive for CAR‐T activity.

Clinical translation of TME‐remodelling strategies: lessons and gaps. Clinical efforts to remodel the TME in conjunction with CAR‐T therapy are yielding initial insights. The combination of intrapleurally delivered mesothelin‐targeted CAR‐T cells with the PD‐1 inhibitor pembrolizumab for malignant pleural mesothelioma demonstrated feasibility, a manageable safety profile, and an encouraging median overall survival of 23.9 months, providing clinical proof‐of‐concept for locoregional delivery combined with checkpoint blockade [158]. Similarly, regional (intraperitoneal or intravenous) delivery of a hypoxia‐responsive, CEA‐targeted CAR‐T cell (PC13) showed manageable toxicity and promising efficacy in a phase I study, with higher objective response rates observed in patients with peritoneal metastases receiving intraperitoneal infusion or those without liver metastases [167]. These studies validate the preclinical principle that local delivery and modulating the TME can enhance therapeutic index. However, strategies involving more complex genetic engineering to directly armour CAR‐T cells with immunomodulatory functions are at earlier stages of clinical testing. A phase I trial of TGFβ‐resistant, PSMA‐targeted CAR‐T cells for prostate cancer demonstrated feasibility and trafficking but limited clinical activity, with CAR‐T cell failure associated with upregulation of multiple inhibitory pathways in the TME, emphasising the redundancy of immunosuppressive mechanisms [168]. The arsenal of preclinical strategies to deplete stromal components or broadly target immunosuppressive cells carries significant translational hurdles, including risks of disrupting normal tissue homeostasis and managing potential systemic toxicities. The path forward requires iterative clinical testing with deep correlative analysis to understand which TME components are dominant in specific patient populations and to develop rational, patient‐stratified combination strategies.

## 6 Clinical Translation: Toxicity, Tracking, and Tailored Applications

Transitioning engineered CAR‐T cells from preclinical success into routine clinical practice entails three interconnected priorities: managing immune‐mediated toxicities such as CRS and neurotoxicity, implementing real‐time in vivo monitoring, and tailoring strategies to the distinct challenges of both solid and hematologic malignancies. Clinical datasets are informing iterative design refinements. Key aspects of toxicity mitigation, monitoring, and indication‐specific optimisation are summarised in Table 5.

**TABLE 5:** Clinical insights and specialised applications of CAR‐T therapy.

CAR‐T type/strategy	Mechanism	Function	Cancer immunotherapy application	References
Microglia‐modulated CAR‐T	Transient microglial depletion/CCR3 blockade	Rescue of oligodendrocyte homeostasis and cognitive function	CNS/non‐CNS cancers with neuroinflammation risk	[169]
BCA management protocol	Monitoring B‐cell precursor reconstitution	Guidance for IVIg replacement timing	CD19 CAR‐T recipients with prolonged B‐cell aplasia	[170]
Fluorescent Reporter CAR‐T	Co‐expression of GFP/luciferase with CAR	Quantitative biodistribution and tumour trafficking kinetics	Preclinical CAR‐T product optimisation	[171]
HDSCA‐HemeCAR Liquid Biopsy	Single‐cell analysis of CAR‐T cells and CD19 epitope landscape	Detection sensitivity 1:3 M cells; CD19 isoform heterogeneity mapping	CD19 CAR‐T recipients for relapse risk stratification	[172]
BCMA CAR‐T	Impaired CAR‐T fitness in prior ASCT recipients	Identification of high‐risk cohort (4‐year OS: 63.2% vs. 82.1%)	RRMM requiring optimised cell product selection	[173]
	4‐year outcome analysis	37.4% PFS; 63.2% OS (ASCT/EMD negative predictors)	R/R multiple myeloma	
BCMA CAR‐T (ide‐cel vs. cilta‐cel)	Comparative CD4and CAR‐T expansion and CD27 dynamics	Differential peak expansion/duration predictive of toxicity	RRMM product selection and toxicity anticipation	[174]
G‐SNAT‐Cy5 Imaging	gzmB‐activated fluorophore aggregation	Real‐time visualisation of cytotoxic T lymphocyte (CTL) cytotoxic activity	Therapy response assessment across immunotherapies	[175]
Allo CD19 CAR‐CIK	Donor‐derived SB‐transposed CIK cells	86% CR/CRi (5/6 MRD‐) at highest dose	Post‐alloHSCT relapsed B‐ALL	[176]
CD30 CAR‐T	Minimal toxicity design	PROs equivalent to healthy population at 4 weeks	CD30and lymphomas	[177]
Intrapleural MSLN CAR‐T and PD‐1i	Regional delivery and checkpoint blockade	Median OS 23.9 months; 83% 1‐year OS	Malignant pleural mesothelioma/lung mets	[158]
MSLN CAR‐T (ovarian)	High‐affinity scFv targeting mesothelin	Tumour sphere elimination in PDX models	Platinum‐resistant ovarian cancer	[178]
MSLN CAR‐T and PPZ	Proton pump inhibition normalising lysosomal pH	Restored membrane MSLN expression	Breast/NSCLC brain metastases	[179]
EGFRvIII CAR‐T and PD‐1i	Tumour‐specific mutation targeting and checkpoint blockade	Limited efficacy (T‐cell exhaustion dominant)	Recurrent glioblastoma	[180]
CLDN18.2 CAR‐T	Tight junction protein targeting	Complete/partial responses	Gastric/pancreatic adenocarcinoma	[106]
GD2 CAR‐T (paediatric solid)	Multi‐omics profiling of TME determinants	Naïve T‐cell/CXCR3and monocyte correlation with expansion	Osteosarcoma/neuroblastoma	[181]
CLL1 CAR‐T (paediatric)	Stem cell antigen targeting	50% MRD‐CR (1–2 grade CRS)	R/R paediatric AML	[113]
BCMA‐BBZ‐OX40 CAR‐T (InstanCART)	OX40 co‐stimulation and rapid manufacturing (3 days)	Comparable efficacy to traditional manufacturing	R/R multiple myeloma	[182]
BCMA/GPRC5D bsCAR	Dual‐antigen targeting	100% ≥ PR; 44.4% CR; 63% 12‐months PFS	RRMM with extramedullary disease	[183]
DARIC33 CAR‐T	Rapamycin‐controlled split CAR	HSC sparing activity	Paediatric R/R AML	[56]
CD19 CAR‐T and ibrutinib	BTK inhibition enhancing T‐cell fitness	80% CR; 75% 12‐months PFS (TP53mut insensitive)	R/R mantle cell lymphoma	[184]
BCMA CAR‐T (AL amyloidosis)	Plasma cell depletion	Hematologic responses and organ function improvement	Systemic light‐chain amyloidosis	[185]

### 6.1 Clinical Toxicity Spectrum and Mitigation Strategies

**FIGURE 9:**
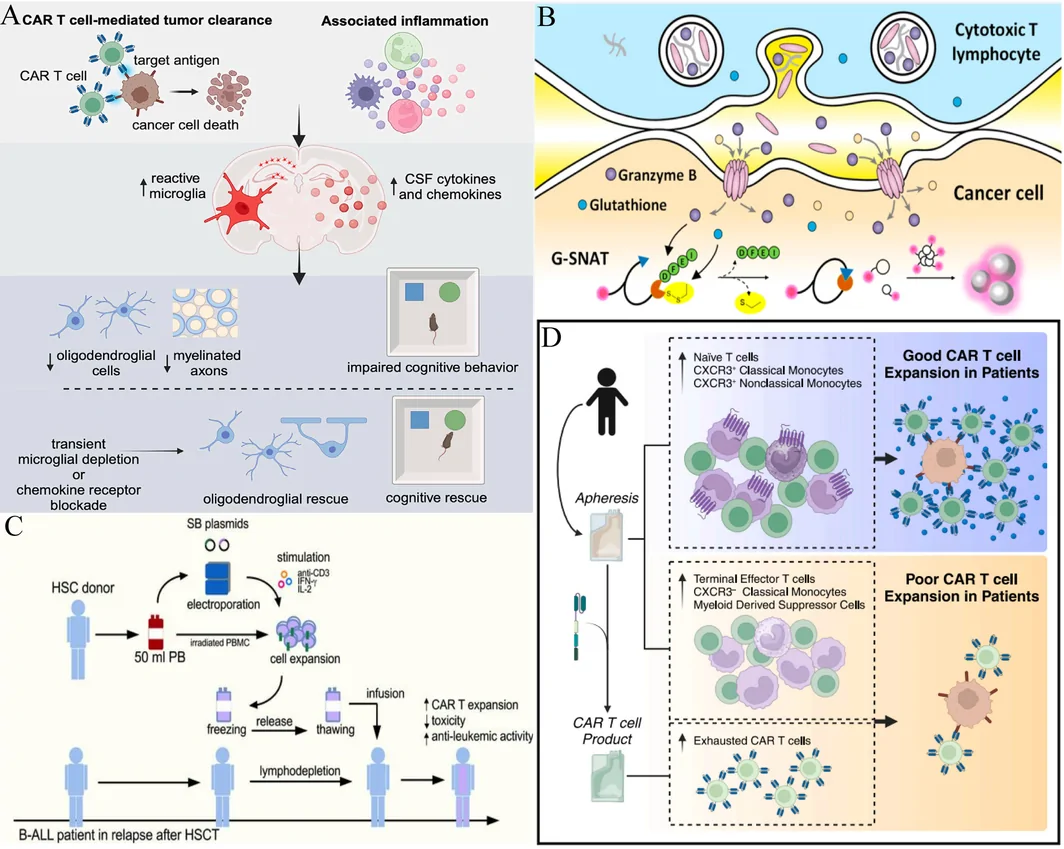
Advanced strategies for characterizing safety, efficacy, and neurotoxicity in CAR‐T cell immunotherapy. (A) Schematic illustration: Immunotherapy‐related cognitive impairment after CAR T cell therapy in mice. Reproduced with permission [169]. Copyright from Elsevier Ltd., 2025. (B) Schematic illustration: Multiparameter longitudinal imaging of immune cell activity in chimeric antigen receptor T cell and checkpoint blockade therapies. Reproduced with permission [172]. Copyright from American Chemical Society, 2022. (C) Schematic illustration: Sleeping beauty‐engineered CAR T cells achieve anti‐leukaemic activity without severe toxicities. Reproduced with permission [176]. Copyright from The American Society for Clinical Investigation (ASCI), 2020. (D) Schematic illustration: Immune determinants of CAR‐T cell expansion in solid tumour patients receiving GD2 CAR‐T cell therapy. Reproduced with permission [181]. Copyright from Elsevier Ltd., 2024.

Cytokine release syndrome (CRS) remains a central target for engineering‐based mitigation strategies. Intracellular checkpoint modulation within CAR‐T cells has shown promise; co‐expression of SOCS1 reduced inflammatory cytokines—70% for IL‐6 and 65% for IFN‐γ—while maintaining cytotoxic activity, improving the therapeutic index in preclinical models [67]. Neurotoxicity poses broader and more complex challenges that extend beyond acute cytokine surges. Preclinical work indicated that sustained microglial activation and oligodendrocyte dysfunction induced cognitive deficits, including attention and memory impairments; transient microglial depletion or CCR3 blockade restored cognitive performance, implicating neuroinflammation as a modifiable mechanism [169] (Figure 9A). Prolonged persistence of functional CAR‐T cells, although desirable for disease control, can lead to sustained B‐cell aplasia (BCA), necessitating monitoring of immunoglobulin levels and management of hypogammaglobulinemia [170].

### 6.2 Advanced Technologies for In Vivo CAR‐T Monitoring

Real‐time monitoring technologies are improving the characterisation of CAR‐T trafficking, biodistribution, and functional states in patients. Liquid biopsy platforms such as the high‐definition single‐cell assay for CAR‐T cells (HDSCA‐HemeCAR) detected rare CAR‐T cells at a sensitivity of 1 in 3 million nucleated cells, enabling assessment of CD19 epitope heterogeneity and identification of antigen escape variants in B‐cell malignancies [171]. Activatable probes, including a granzyme B‐sensitive nano‐assembly tracer conjugated to Cy5 (G‐SNAT‐Cy5), reported granzyme B activity via intramolecular cyclisation upon cleavage, providing higher spatial resolution of cytotoxic engagement than conventional bioluminescence [172] (Figure 9B). Longitudinal analysis clarified organ‐specific sequestration and expansion kinetics. In BCMA CAR‐T studies, prior autologous stem cell transplantation (ASCT) and extramedullary disease correlated with a 45% reduction in peak expansion [173], indicating that product and host factors shape outcomes.

The integration of trackable reporters directly into the CAR‐T cell genome via CRISPR‐Cas9 editing enables precise, long‐term monitoring. The tRACE‐CAR system allows for the site‐specific integration of imaging reporter genes alongside the CAR construct. This platform enabled non‐invasive, dynamic tracking of CAR‐T cell distribution and persistence in leukaemia and ovarian cancer models, revealing how administration routes (intravenous vs. intraperitoneal) significantly impact tumour homing and therapeutic efficacy [186]. Beyond tracking location, understanding clonal dynamics is crucial. Combined single‐cell T cell receptor (TCR) and CAR sequencing technologies now allow researchers to simultaneously track the fate of endogenous T cell clones and engineered CAR‐T products, providing unprecedented insight into their competitive or cooperative interactions within the tumour immune microenvironment [187].

Comparative product analyses have revealed distinct clinical and immunological profiles. Ciltacabtagene autoleucel (cilta‐cel) was associated with greater proliferation of CD4^+^ CAR‐T cells and higher CD27 expression—both markers of persistence—compared with idecabtagene vicleucel (ide‐cel), but also with higher rates of neurotoxicity (25% vs. 9%) and infection, necessitating proactive risk management [174]. Sustaining long‐term immune memory and preventing recurrence remain priorities. In multiple myeloma, relapse rates ranged from 40% to 60% over 2 years post–CAR‐T therapy [175], reflecting antigen loss, T‐cell exhaustion, and persistent TME suppression. Extended follow‐up of BCMA CAR‐T therapy in 141 relapsed/refractory patients documented an overall response rate of 94.8% and a complete response rate of 50.7%, with four‐year progression‐free and overall survival of 37.4% and 63.2%, respectively [173].

Monitoring has broadened across indications and platforms. Donor‐derived CD19 CAR cytokine‐induced killer (CIK) cells engineered by the Sleeping Beauty transposon achieved durable remissions, with an 85.7% CR/CRi rate at the maximum dose and a 71.4% MRD‐negative CR in B‐ALL relapsing after allogeneic HSCT [176] (Figure 9C). CD30‐targeted CAR‐T therapy was associated with favourable patient‐reported outcomes, with symptom burden and physical functioning returning to or exceeding baseline within 4 weeks post‐infusion, indicating manageable toxicity [177].

### 6.3 Clinical Translation and Engineering Lessons for Solid Tumours

Translating integrated engineering strategies into clinical practice for solid tumours requires adapting lessons from the more advanced field of hematologic malignancies while addressing unique solid tumour barriers. The clinical experience in blood cancers has validated core engineering principles—such as the importance of co‐stimulation domains, managing cytokine release syndrome (CRS), and countering antigen escape—which now serve as a foundation for solid tumour efforts. However, the distinct challenges of the solid TME, including poor trafficking, immunosuppression, and metabolic stress, demand additional, specialised engineering layers. Here, we discuss the clinical translation of integrated approaches for solid tumours, contextualised by relevant insights from hematologic CAR‐T therapy. Regional administration has improved control in confined sites. In MPM, intrapleural delivery of MSLN‐targeted CAR‐T cells combined with pembrolizumab achieved a median overall survival of 23.9 months; CAR‐T persistence beyond 100 days was observed in 39% of patients, with some attaining complete metabolic responses [158]. Intraperitoneal delivery has also been implemented in ovarian cancer [157, 178]. TME‐driven antigen loss remains a key constraint; in brain metastases with acidic microenvironments, reduced MSLN surface expression limited efficacy. Pantoprazole elevated intratumoural pH, inhibited lysosomal degradation, and increased antigen expression, thereby enhancing CAR‐T cytotoxicity [179]. Persistent immunosuppression continues to limit certain approaches; EGFRvIII CAR‐T cells combined with PD‐1 blockade showed modest activity in recurrent glioblastoma [180]. Ongoing target validation has yielded clinical signals, with CLDN18.2–directed CAR‐T therapy demonstrating activity, including complete responses, in advanced gastric and pancreatic cancers [106]. GD2 CAR‐T therapy has been explored in osteosarcoma and neuroblastoma, where specific T‐cell and monocyte subsets correlated with expansion kinetics [181] (Figure 9D). The clinical translation of integrated engineering strategies for solid tumours is providing critical lessons. Beyond CLDN18.2, other targets are being actively explored. A phase I trial of GD2‐CAR T cells in combination with BRAF/MEK inhibitors for metastatic melanoma and as monotherapy for other solid tumours confirmed feasibility and safety but showed limited clinical efficacy, underscoring the need for more potent engineering beyond target engagement alone [188]. For recurrent high‐grade glioblastoma, intracerebroventricular administration of B7‐H3‐targeted CAR T cells (BrainChild‐03 trial) was well‐tolerated with repeated dosing and associated with a median overall survival from diagnosis of 19.8 months, suggesting potential clinical activity for localised CNS delivery [189]. Innovative engineering approaches are entering the clinic. A phase I trial of CTX130, an allogeneic CD70‐targeting CAR T‐cell product for clear cell renal cell carcinoma, reported a manageable safety profile, disease control in 81.3% of patients, and one durable complete response lasting over 3 years, providing proof‐of‐concept for allogeneic CAR‐T in solid tumours [190]. Similarly, a first‐in‐human trial of the ‘off‐the‐shelf’ allogeneic B7‐H3‐targeted CAR‐Vδ1 T cell product UTAA06 demonstrated a manageable profile with no GvHD but limited persistence and efficacy, attributed to host‐versus‐graft rejection, highlighting a key challenge for universal products [191]. Engineering to enhance potency is also being tested clinically. Co‐expression of IL‐15 in GPC3‐targeted CAR T cells (15.CAR) significantly increased cell expansion and improved the antitumour response rate compared to non‐armoured CAR‐T cells in patients with hepatocellular carcinoma, validating the concept of cytokine engineering, though it was associated with increased cytokine release syndrome [38]. These studies collectively highlight that while regional delivery and target selection have shown the most clinical activity to date, challenges of persistence, immunosuppression, and host rejection (for allogeneic products) remain significant barriers. The integration of more sophisticated engineering—such as cytokine arming, logic gating, and strategies to overcome the allogeneic barrier—into well‐designed clinical trials with robust biomarker analyses will be essential to determine if these approaches can translate preclinical promise into durable clinical responses.

Recent clinical studies continue to inform engineering strategies. In paediatric B‐ALL, a fully human tandem CD19/CD22 CAR (CAR22.19) demonstrated a manageable safety profile and promising activity, including in patients with CD19‐negative disease, highlighting the utility of dual‐targeting to prevent antigen escape. However, limited in vivo persistence was noted as a potential factor in some relapses, underscoring that enhancing durability remains a critical engineering goal beyond target engagement [192]. Furthermore, the application of CAR‐T therapy is expanding beyond oncology. Dual anti‐CD19/anti‐BCMA CAR‐T cells have shown remarkable efficacy and a favourable safety profile in patients with treatment‐refractory systemic lupus erythematosus (SLE), inducing deep and sustained remission by targeting both autoreactive B cells and long‐lived plasma cells. This success in autoimmunity validates the potency of CAR‐T technology and opens new avenues for its application [193]. In another autoimmune application, a CD19/CD70 dual CAR‐T strategy was designed to simultaneously eliminate pathogenic B cells and alloreactive T cells, potentially enabling the use of allogeneic CAR‐T products with less intensive lymphodepletion [194].

In hematologic malignancies, engineering focuses on refining efficacy and countering resistance. CLL1‐targeted CAR‐T cells achieved a morphological leukaemia‐free state and MRD negativity in 50% (4/8) of paediatric relapsed/refractory AML cases, with manageable CRS [113]. Co‐stimulatory optimisation was reflected in BCMA‐BBZ CAR‐T cells incorporating an independent OX40 domain, which conferred enhanced cytotoxicity and reduced exhaustion [182]. Bispecific designs such as BCMA/GPRC5D CAR‐T cells achieved a 100% overall response rate in relapsed/refractory multiple myeloma [183]. Pharmacologically regulated systems, including the rapamycin‐dependent DARIC33 platform, were configured to protect haematopoietic stem and progenitor cells (HSPCs) and expand the therapeutic window in paediatric relapsed/refractory AML [56]. Rational combinations further improved outcomes; ibrutinib plus CD19 CAR‐T therapy yielded an 80% complete response rate in mantle cell lymphoma [184]. Application has broadened beyond myeloma, with BCMA CAR‐T cells inducing responses in systemic light‐chain amyloidosis [185].

## 7 Multiscale Technologies and Precision Engineering Converging to Advance CAR‐T Therapy in Solid Tumours

Progress in CAR‐T‐cell therapy for solid tumours is being increasingly accelerated by advanced analytical platforms that resolve spatiotemporal interactions between effector cells and the TME, moving beyond static endpoint assessments toward mechanistic insights that directly inform rational product design. High‐throughput Bessel light‐sheet oblique plane microscopy enabled real‐time, three‐dimensional analysis of immune synapse formation, quantifying biophysical parameters such as actin flow and synaptic contact area that correlated with cytotoxic potency [195]. At the nanoscale, single‐molecule localisation microscopy has revealed that CARs—including CD138‐directed constructs—undergo ligand‐triggered self‐aggregation and segregate more effectively from the inhibitory phosphatase CD45 upon cancer cell engagement, with the degree of nanoscale organisation correlating with calcium influx and target‐cell killing [196]. These observations align with evidence that CAR molecular architecture and membrane organisation—shaped by co‐stimulatory domains and scaffold elements—govern clustering and diffusion dynamics that predict synapse quality and early activation. Integrating these biophysical determinants into construct selection is critical to minimise tonic signalling and maximise productive synapse formation [197].

**FIGURE 10:**
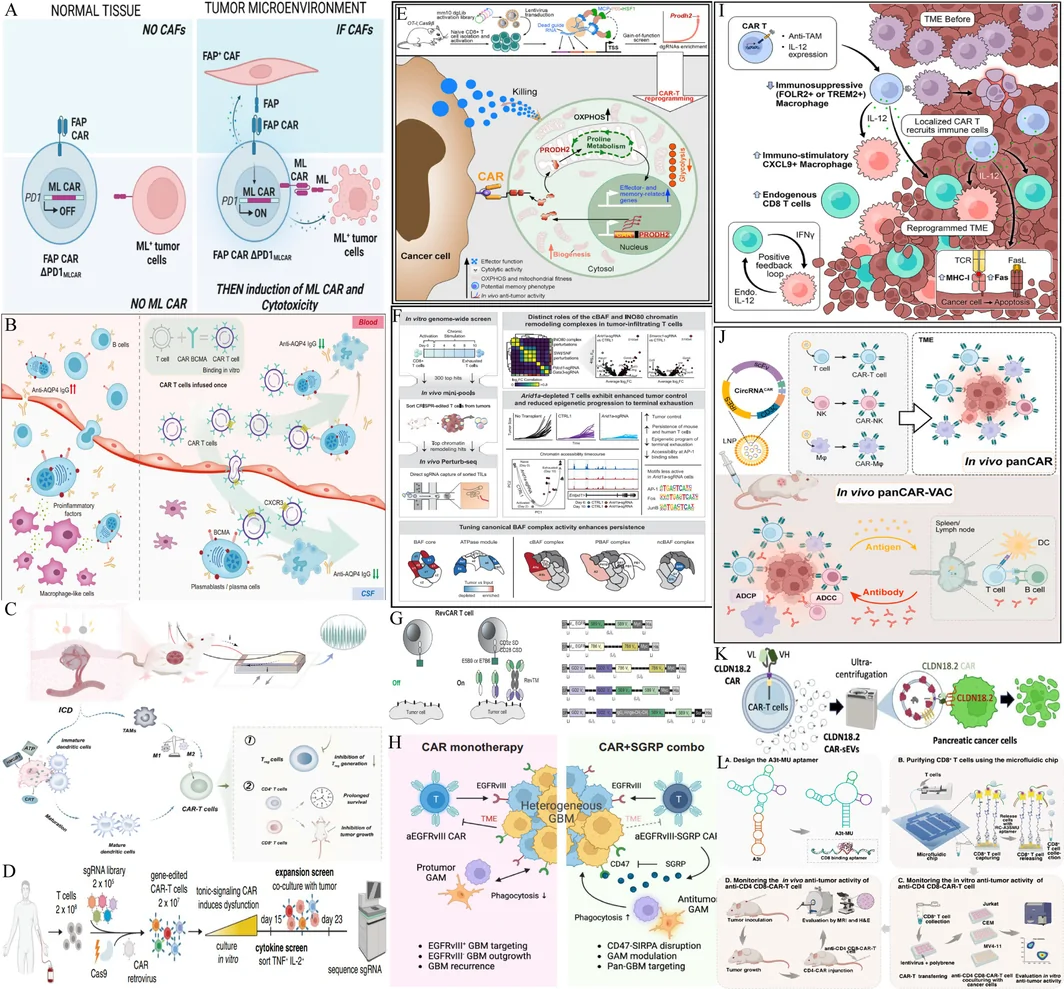
Next‐Generation genome engineering and multi‐targeting architectures for optimizing CAR‐T cell persistence, controllability, and therapeutic reach. (A) Schematic illustration: Ranscription activator‐like effector nuclease (TALEN)‐medited allogeneic inducible dual CAR T‐cells enable effective targeting of solid tumours while mitigating off‐tumour toxicity. Reproduced with permission [198]. Copyright from Elsevier Ltd., 2024. (B) B‐cell maturation antigen (BCMA)‐targeted CAR T‐cell therapy effectively reverses immune dysfunction within the cerebrospinal fluid (CSF) of patients with neuromyelitis optica spectrum disorder (NMOSD), a condition in which the accumulation of clonally expanded plasmablasts (PBs) and plasma cells (PCs) correlates positively with neuroinflammation and subsequent neural injury. Engineered anti‐BCMA CAR T cells, characterized by enhanced chemotactic properties and upregulated CXCR3 expression, efficiently traverse the blood‐CSF barrier to deplete these pathogenic PB/PC populations, thereby suppressing myeloid cell activation and attenuating neuroinflammation in the NMOSD clinical setting. Reproduced with permission [199]. Copyright from The American Association for the Advancement of Science, 2024. (C) Schematic diagram depicts the enhancement of CAR‐T cell therapy for solid tumours via triboelectric immunotherapy‐mediated reprogramming of the immunosuppressive microenvironment. Reproduced with permission [200]. Copyright from Elsevier Ltd., 2025. (D) Schematic representation of a clustered regularly interspaced short palindromic repeats (CRISPR) knockout screen identifying modulators of cytokine secretion and CAR T‐cell proliferation within a tonic signalling‐induced exhaustion model. Reproduced with permission [201]. Copyright from The American Association for the Advancement of Science, 2022. (E) Schematic illustration: A genome‐scale gain‐of‐function CRISPR screen in CD8 T cells identifies proline metabolism as a means to enhance CAR‐T therapy. Reproduced with permission [202]. Copyright from Elsevier Ltd., 2022. (F) Schematic illustration: Genome‐wide CRISPR screens of T cell exhaustion identify chromatin remodelling factors that limit T cell persistence. Reproduced with permission [203]. Copyright from Elsevier Ltd., 2022. (G) Schematic representation of the reverse chimeric antigen receptor (RevCAR) system, wherein RevCAR T cells express a reverse chimeric antigen receptor composed of intracellular CD3ζ signalling (SD) and CD28 co‐stimulatory domains (CSD), a CD28 transmembrane domain, a hinge, and extracellular peptide epitopes (E5B9 or E7B6), with activation occurring exclusively upon bispecific targeting module for RevCAR (RevTM)‐mediated redirection to tumour cells. The corresponding bispecific RevTMs primarily comprise tumour‐associated antigen (TAA)‐specific and epitope‐specific variable domains—derived from monoclonal antibodies (V_H_, V_L_) or camelid nanobodies and joined by peptide linkers—or alternatively incorporate human IgG4 hinge and constant regions (C_H_2, C_H_3). To facilitate secretion, detection, and purification, all molecules include an N‐terminal signal peptide (SP) and C‐terminal Myc and 6× Histidine (His) tags, the latter of which can be removed via an upstream sortase cleavage site (Sor) if required. Reproduced with permission [204]. Copyright from Frontiers Media SA, 2023. (H) Mechanism of action of conventional epidermal growth factor receptor variant III (EGFRvIII) CAR T cell monotherapy in GBM. Reproduced with permission [205]. Copyright from Springer Nature, 2024. (I) Schematic illustration: Armoured macrophage‐targeted CAR‐T cells reset and reprogram the tumour microenvironment and control metastatic cancer growth. Reproduced with permission [41]. Copyright from Elsevier Ltd., 2026. (J) Schematic illustration: Synergically enhanced anti‐tumour immunity of in vivo panCAR by circRNA vaccine boosting. Reproduced with permission [206]. Copyright from Elsevier Ltd., 2025. (K) Schematic representation of CAR‐engineered small extracellular vesicle (CAR‐sEV) secretion from Claudin 18.2 (CLDN18.2) CAR‐T cells. Reproduced with permission [207]. Copyright from John Wiley and Sons, 2025. (L) Schematic illustration: Efffcient capture and traceless release of functional CD8^+^ T cells with a microffuidic chip for enhanced in vitro and in vivo CD4‐CAR transduction. Reproduced with permission [208]. Copyright from American Chemical Society, 2024.

Accurate forecasting of clinical outcomes also requires situating CAR‐T‐cell function within its proper tissue context. Spatial multi‐omics disentangled cellular topology and signalling gradients that condition response. In large B‐cell lymphoma, pre‐treatment spatial profiling distinguished complete responders by enriched immune infiltration and T‐cell migration programs, whereas non‐responders showed T‐cell and macrophage dysfunction, including reduced separation between PD‐1^+^ T cells and PD‐L1^+^ cells that provided a spatial substrate for suppression [198] (Figure 10A). In non‐small cell lung cancer, analyses after neoadjuvant therapy indicated that the ratio of tertiary lymphoid structure area to tumour area discriminated responders, and that the distance between PD‐L1^+^CD11c^+^ cells and PD1^+^CD8^+^ T cells within these structures varied with treatment modality, highlighting spatial regulation of immunity [209]. Single‐cell multi‐omics complemented tissue‐scale context by delineating CAR‐T‐cell heterogeneity and determinants of persistence. In multiple myeloma, pre‐infusion profiling identified a pre‐existing suppressive milieu in non‐responders, marked by increased CD39^+^ monocytes that attenuated CD8^+^ and natural killer (NK) cell function and by expanded CAR‐T‐cell clones with cytotoxic yet exhausted phenotypes [210]. In neuromyelitis optica spectrum disorder, proliferating cytotoxic‐like CD8^+^ CAR‐T‐cell clones emerged as dominant effectors but exhibited a restrained cytotoxic signature compared with hematologic malignancies, indicating disease‐specific effector programs [199] (Figure 10B). These datasets indicate that efficacy is co‐determined by engineered attributes and the dynamic host immune milieu.

Discovery insights are translating into precision engineering. A primary barrier—on‐target/off‐tumour toxicity—is being addressed by epitope‐specific editing. Optimised prime editing reconfigured the CD123 epitope in HSPCs to impair CAR binding while preserving protein function, opening a therapeutic window for AML [200] (Figure 10C). Adenine base editing of the CAR‐recognised epitope on CD45 installed function‐preserving mutations that permitted CD45‐directed CAR‐T‐cell activity against leukaemias without compromising haematopoiesis, as edited HSPCs retained engraftment and differentiation [211]. Durability and potency have been systematically improved using genome‐wide CRISPR platforms. Screens identified SOCS1 as an intrinsic checkpoint limiting the expansion and polyfunctionality of antigen‐experienced CD4^+^ T cells; its inactivation enhanced intratumoural persistence and function [67]. The Mediator kinase module (e.g., MED12, CCNC) acted as a brake on effector activity; genetic deletion or pharmacologic inhibition augmented proliferation and antitumour efficacy [201] (Figure 10D). CRISPR activation screens implicated PRODH2, a proline metabolism enzyme, whose upregulation reshaped transcriptional and metabolic states to improve tumour control [202] (Figure 10E). Epigenetic mapping highlighted regulators of exhaustion, including INO80 and BAF complex subunits, suggesting that chromatin remodelling can sustain effector programs and prolong persistence [203] (Figure 10F).

These principles inform next‐generation cellular architectures tailored to solid tumours. Logic‐gated platforms such as the Reversible and Versatile CAR (RevCAR) system, which uses bispecific, switchable modules to activate CAR‐T cells only in the presence of dual antigens, increasing specificity and adding safety control [204] (Figure 10G). A single‐vector synNotch system embedded complex gating within one lentiviral construct, achieving high specificity and antitumour activity with minimal baseline toxicity in preclinical models [212]. Surface engineering refined fitness; fusing the CTLA‐4 cytoplasmic tail to the CAR increased endocytosis, reduced trogocytosis and antigen loss, enhanced cytotoxicity, and promoted a central memory–like state in recurrent leukaemia models [213]. Effector cells were also armed to remodel suppressive niches: CAR‐T cells secreting a high‐affinity CD47 blocker simultaneously killed glioblastoma cells and neutralised the CD47–SIRPα axis, reinvigorating macrophage and microglial phagocytosis [205] (Figure 10H). Targeting TAMs and incorporating IL‐12 expression durably reprogrammed the TME, expanding CXCL9^+^ macrophages and endogenous tumour‐specific cytotoxic T cells that persisted beyond CAR‐T contraction [41] (Figure 10I).

The engineering horizon continues to expand, encompassing alternative cell sources and innovative delivery formats to improve patient access, product consistency, and therapeutic safety. Allogeneic ‘off‐the‐shelf’ products advanced through multiplex editing with orthogonal CRISPR systems, coupling SaCas9‐mediated base edits that disrupt B2M and REGNASE‐1 with SpCas9‐directed CAR integration at TRAC, reducing risks associated with double‐strand break–dependent approaches while preserving potency [214]. Umbilical cord blood–derived T cells provided an alternative source with reduced exhaustion, a higher memory fraction, and superior antitumour activity compared with peripheral blood–derived counterparts in preclinical models [215]. In vivo and cell‐free strategies are emerging. A circRNA‐based platform generated pan‐CAR immune cells—including CAR‐T, CAR‐NK, and CAR macrophages—within the host, suppressing tumour growth and reshaping the TME, particularly when combined with a circRNA vaccine [206] (Figure 10J). Small extracellular vesicles derived from CAR‐T cells, such as CLDN18.2‐directed vesicles, produced antitumour effects in pancreatic ductal adenocarcinoma models without systemic cytokine release, indicating a modular cell‐free modality [207] (Figure 10K). Manufacturing and monitoring innovations are streamlining translation: functionalised microfluidic chips with optimised aptamers enriched CD8^+^ T cells to yield more homogeneous and potent products [208] (Figure 10L); digital nanoplasmonic microarray immunosensors enabled multiplex, time‐resolved tracking of cytokine dynamics to guide efficacy and toxicity management, including CRS [216]; and integrative models such as Inflammatory Signature Mixture (InflaMix) combined pre‐infusion laboratory parameters with cytokine metrics to improve prediction of treatment failure in non‐Hodgkin lymphoma [217]. The pursuit of universal, off‐the‐shelf CAR products is intensifying, with a focus on overcoming host rejection. Beyond multiplex gene editing to disrupt TCR and human leukocyte antigen (HLA) molecules, novel ‘stealth’ strategies are emerging. Engineering allogeneic CAR‐T cells to overexpress CD47, a ‘don't eat me’ signal, has been shown to protect them from macrophage‐ and neutrophil‐mediated phagocytosis, significantly improving their persistence and antitumour efficacy in immunocompetent hosts [70]. The clinical pipeline for allogeneic therapies is expanding, with products derived from healthy donor peripheral blood mononuclear cells (PBMCs), cord blood, or induced pluripotent stem cells (iPSCs) advancing through trials [218]. However, mitigating host versus graft rejection (HvGR) remains a critical hurdle for long‐term persistence, driving research into strategies such as additional HLA editing or the use of cell sources with low intrinsic alloreactivity, like γδ T cells [219, 220]. Simultaneously, epigenetic pharmacologic modulation is showing clinical translational promise. In an ovarian cancer trial, the combination of mesothelin‐targeted CAR‐T cells with the histone deacetylase inhibitor sodium valproate (VPA) enhanced CAR‐T cell cytotoxicity, reduced exhaustion, and improved tumour infiltration. Mechanistically, VPA induced histone propionylation, leading to transcriptional activation of genes like LOX (promoting migration) and GUCY1B3 (enhancing metabolic fitness), illustrating a viable path to clinically augment CAR‐T function through metabolic‐epigenetic reprogramming [144].

These multiscale technologies—from subcellular imaging and spatial multi‐omics to precise genome editing, logic‐gated cell engineering, and innovative delivery systems—are moving CAR‐T development from trial‐and‐error toward rational design. By coupling synapse‐level biophysics with tissue topology and incorporating programmable safety and fitness modules, next‐generation CAR‐T cells are better positioned to navigate and overcome layered barriers within immunosuppressive solid TMEs, with the aim of achieving durable and safe clinical benefit. Table 6 provides a systematic summary of these technologies and engineering strategies, delineating mechanisms, functional impacts, and applications in solid tumour immunotherapy.

**TABLE 6:** Multiscale profiling and precision engineering strategies advancing CAR‐T therapy in solid tumours.

Technology/platform/strategy	Category/mechanism	Primary function/application	Impact on tumour immunotherapy	References
High‐throughput Bessel light‐sheet oblique plane microscopy (HBOPM)	Imaging and spatiotemporal profiling	Enables real‐time, 3D analysis of immune synapse formation; quantifies actin flow and synaptic contact area.	Correlates biophysical synapse parameters with cytotoxic potency, informing CAR construct design.	[195]
Single‐molecule localisation microscopy (SMLM)	Imaging and spatiotemporal profiling	Visualises nanoscale CAR clustering and segregation from CD45 upon target engagement.	Links CAR nanoscale organisation with effector functions (calcium influx, killing), guiding molecular engineering.	[196]
Biophysical analysis of CAR architecture	Imaging and spatiotemporal profiling	Investigates how co‐stimulatory domains and scaffold elements govern CAR clustering and diffusion dynamics.	Predicts synapse quality and early activation; critical for minimising tonic signalling and maximising productive engagement.	[197]
Spatial multi‐omics (lymphoma application)	Spatial profiling and multi‐omics	Analyses pre‐treatment tissue architecture, immune infiltration, and cell–cell proximity (e.g., PD‐1and/PD‐L1and).	Distinguishes responders from non‐responders; reveals spatial basis for immune suppression.	[198]
Spatial multi‐omics (NSCLC application)	Spatial profiling and multi‐omics	Measures tertiary lymphoid structure area and distances between immune subsets post‐neoadjuvant therapy.	Identifies spatial biomarkers of response and highlights treatment‐modulated immune regulation.	[209]
Single‐cell multi‐omics (multiple myeloma)	Single‐cell profiling	Profiles pre‐infusion TME and CAR‐T clones, identifying suppressive CD39and monocytes and exhausted CAR‐T phenotypes.	Predicts non‐response by revealing a pre‐existing immunosuppressive landscape.	[210]
Single‐cell profiling (neuromyelitis optica spectrum disorder (NMOSD) application)	Single‐cell profiling	Characterises dominant effector CAR‐T clones (proliferating cytotoxic‐like CD8and) and their transcriptional signature.	Reveals disease‐specific CAR‐T effector programs, informing tailored engineering.	[199]
SOCS1‐knockout CAR‐T (CD4and)	Genome engineering and fitness enhancement	CRISPR‐Cas9 inactivation of the SOCS1 checkpoint in CD4and T cells.	Enhances intratumoural accumulation, proliferation, survival, and polyfunctionality of CD4and CAR‐T cells.	[67]
PRODH2‐overexpressing CAR‐T	Genome engineering and fitness enhancement	CRISPR activation (CRISPRa)‐driven overexpression of the proline metabolism enzyme PRODH2.	Reprograms metabolism and transcriptome; enhances CAR‐T cell antitumour efficacy in vitro and in vivo.	[202]
Mediator kinase module inhibition (e.g., MED12/CCNC KO)	Genome engineering and fitness enhancement	Genetic deletion or pharmacological inhibition of the Mediator kinase module.	Releases transcriptional repression, enhancing CAR‐T cell proliferation and antitumour activity.	[201]
Epigenetic regulator knockout (e.g., INO80/BAF)	Genome engineering and fitness enhancement	CRISPR knockout of epigenetic regulators (e.g., INO80, BAF complex subunits).	Sustains effector programs and delays exhaustion, improving CAR‐T cell persistence.	[203]
Epitope editing of CD45 via base editing	Genome engineering and safety	Adenine base editing to install function‐preserving mutations in the CD45 epitope on HSPCs.	Enables safe targeting of pan‐leukocyte CD45 by CAR‐T cells without ablating haematopoiesis.	[211]
Epitope editing of CD123 via prime editing	Genome engineering and safety	Optimised prime editing to alter the CD123 epitope on HSPCs.	Impairs CAR‐T binding to healthy HSPCs while preserving protein function, mitigating on‐target/off‐tumour toxicity in AML.	[200]
Logic‐gated RevCAR platform	Cellular engineering and safety	Modular, split CAR system activated by bispecific targeting modules requiring dual‐antigen engagement.	Provides AND‐gate logic to enhance tumour specificity and add a safety switch for solid tumours.	[204]
CTLA‐4 cytoplasmic tail (CCT)‐fused CAR	Cellular engineering and fitness	Fusion of CTLA‐4 cytoplasmic tail to CAR endodomain to modulate surface expression via endocytosis.	Reduces trogocytosis/antigen loss, enhances cytotoxicity, and promotes a central memory–like phenotype.	[213]
Digital nanoplasmonic microarray immunosensor	Monitoring and manufacturing	Enables multiplex, time‐resolved tracking of cytokine secretion dynamics.	Informs on efficacy and toxicity management (e.g., CRS) during therapy.	[216]
Aptamer‐based microfluidic CD8and T‐cell selection	Monitoring and Manufacturing	Functionalised microfluidic chip using optimised aptamers for high‐purity selection of CD8and T cells.	Aims to produce more homogeneous and potent CD8and CAR‐T cell products.	[208]
Predictive model InflaMix	Monitoring and Manufacturing	Integrates pre‐infusion laboratory and cytokine metrics to predict outcomes.	Identifies patients at high‐risk of CAR‐T treatment failure in non‐Hodgkin lymphoma.	[217]
CAR‐T cell‐derived small extracellular vesicles (sEVs)	Delivery platform and modality	sEVs derived from CAR‐T cells (e.g., CLDN18.2‐CAR‐T) mediating antigen‐specific killing.	Offers a cell‐free, potentially safer immunotherapy platform with reduced risk of cytokine release.	[207]
circRNA‐based in vivo Pan‐CAR immune cell generation	Delivery platform and modality	In vivo delivery of circRNA vectors to generate CAR‐T, CAR‐NK, and CAR macrophages.	Provides an in vivo engineering platform for multi‐lineage immunotherapy, combinable with vaccines.	[206]
Multiplex‐edited allogeneic CAR‐T (Orthogonal CRISPR)	Cellular engineering and manufacturing	Combines SaCas9 base editing (KO B2M and REGNASE‐1) with SpCas9 for TRAC‐CAR integration.	Generates potent ‘off‐the‐shelf’ allogeneic CAR‐T with reduced risk of genomic rearrangements.	[214]
Umbilical cord blood (UCB)‐derived CAR‐T	Cellular source and manufacturing	Sourcing T cells from umbilical cord blood instead of peripheral blood.	Exhibits reduced exhaustion, higher memory fraction, and superior antitumour activity in preclinical models.	[215]
Single‐vector synNotch (svsNotch) CAR‐T	Cellular engineering and safety	Embeds complex synNotch‐based logic‐gating circuits into a single lentiviral vector.	Achieves superior tumour specificity and efficacy with minimal baseline toxicity in preclinical models.	[212]
SIRPα‐Fc (SGRP)‐secreting CAR‐T (glioblastoma)	Cellular engineering and TME remodelling	CAR‐T cells engineered to secrete a high‐affinity CD47 blocker.	Reinvigorates phagocytosis by macrophages/microglia via CD47‐SIRPα axis blockade, enhancing tumour clearance.	[205]
IL‐12‐secreting, TAM‐targeting CAR‐T	Cellular engineering and TME remodelling	CAR‐T cells targeting tumour‐associated macrophages (TAMs) and expressing IL‐12.	Depletes immunosuppressive TAMs and durably remodels TME, engaging endogenous antitumour immunity.	[41]

The convergence of AI and machine learning with cellular engineering holds significant potential to accelerate and mitigate risks in the development of next‐generation CAR‐T therapies. This synergistic relationship is poised to transform every stage of the therapeutic pipeline. For target discovery, deep learning models analysing multi‐omics datasets can predict novel tumour‐specific antigens, addressing a fundamental bottleneck for solid tumours. In a parallel realm, AI is revolutionising our understanding of disease mechanisms; for instance, in cardiometabolic research, AI is enabling precision diagnostics, biomarker discovery, and patient stratification for cardiovascular diseases [221]. This principle is directly transferable to immunotherapy, where AI models could integrate longitudinal cytokine, metabolic, and imaging data to decode the complex dynamics of the tumour microenvironment and predict CAR‐T cell fate. Beyond target discovery, AI provides a framework for predicting and mitigating toxicity. Machine learning algorithms integrating pre‐infusion patient characteristics with early post‐infusion signalling dynamics can forecast severe CRS or ICANS, enabling preemptive interventions. Furthermore, in patient response stratification, AI‐driven analysis of high‐dimensional data from spatial transcriptomics and radiomics can identify predictive biomarkers, facilitating personalised treatment selection. For instance, understanding the nuanced interplay between metabolic states and immune function—a key insight from studies on AI in cardiac metabolism—can help predict which patients might benefit from metabolically reprogrammed CAR‐T cells versus those requiring cytokine‐armoured constructs. As these datasets grow in size and complexity, the development of transparent, accountable, and collaborative AI models will be critical to ensure equitable access and clinical trust, moving beyond black‐box solutions toward interpretable and actionable insights that guide the data‐driven creation of safer, more effective, and more predictable integrated cellular therapies.

## 8 Discussion

The trajectory of CAR‐T‐cell therapy from hematologic indications to solid tumours necessitates a fundamental shift in perspective, moving from a focus on target‐centric cytotoxicity toward managing the complex interplay between engineered cells and the immunosuppressive TME. This review integrates three interconnected domains: (i) constructing CAR‐T cells with intrinsic resilience, (ii) selecting targets with sufficient discrimination to spare healthy tissues, and (iii) dismantling TME barriers. Durable efficacy in solid tumours is unlikely to arise from a single modality; rather, it depends on combining these axes to produce cellular therapies that are adaptable, persistent, and precise.

Addressing solid tumour barriers necessitates coordinated, multilayered interventions. Contemporary designs prioritise spatiotemporal control beyond structural optimisation, using pharmacologically regulated CARs and stimuli‐responsive platforms to enhance tumour‐restricted activation and limit on‐target/off‐tumour effects [34, 35, 46, 56]. Conventional constructs fail in solid tumours through sequential hurdles: imprecise activation, metabolic and epigenetic vulnerability, antigen heterogeneity and loss, and profound immunosuppression [222]. Precision control therefore needs to be coupled with intrinsic fitness. Epigenetic programming, such as FOXO1 overexpression, reinforced memory‐associated chromatin states, whereas metabolic rewiring with DCA shifted bioenergetics toward OXPHOS, supporting survival under nutrient‐poor, hypoxic conditions [49, 58, 223]. Insufficient mitochondrial fitness contributes to failure; targeting metabolic–immune checkpoints like P4HA1, which accumulates in exhausted T cells and impairs energy production, restored cellular competence in preclinical studies [59, 224]. Optimising intracellular regulators further refined safety, as co‐expression of SOCS1 curtailed inflammatory cytokine output and mitigated CRS while maintaining activity [67, 225].

Remodelling the TME to enable immune activity is equally important. Strategies include depleting suppressive populations such as M2‐polarised TAMs and Tregs [226]. Engineered CAR‐T cells that secrete Sirf CAR‐T blocked CD47‐mediated ‘don't eat me’ signalling, reprogrammed the myeloid compartment, and improved antitumour function [53]. Given redundant suppressive circuits, exemplified by VISTA overexpression in IFN‐γ–poor niches, combinatorial targeting is warranted [148]. Supportive interventions sustained CAR‐T function within adverse TMEs: localised cytokine strategies (e.g., IL‐12, IL‐15) and ‘fitness backpacks’ such as CMC‐21 nanoparticles mitigated acidity and hypoxia [29, 126, 227, 228]. Biomaterial platforms, including GelMA hydrogels and lymph node–mimetic scaffolds, provided spatial and trophic support to improve local retention and persistence [151, 153]. Cellular polarisation and adjuvancy also contributed; Th9‐programmed CAR‐T cells improved persistence, and the secretion of flagellin helped convert immunologically ‘cold’ settings [133, 150].

Strategies to remodel the TME—ranging from cytokine engineering to stromal disruption—aim to convert an immunologically ‘cold’ tumour into a ‘hot,’ immune‐supportive microenvironment. While promising in preclinical models, their translation highlights a critical balance between potency and safety. Armoring CAR‐T cells with potent immunomodulators or enabling them to degrade extracellular matrix or deplete stromal cells can dramatically enhance anti‐tumour activity in mice. However, in patients, this enhanced potency must be carefully controlled to avoid severe on‐target/off‐tumour effects, systemic cytokine release, or damage to normal tissue architecture. The clinical experience with such advanced constructs, while still early, underscores that the human TME is more complex, heterogeneous, and redundantly immunosuppressive than most preclinical models capture. Therefore, the next generation of TME‐modulating strategies must prioritise context‐dependent activation and localised activity. This can be achieved by integrating stricter environmental sensors, leveraging biomaterials for sustained local release, and developing more predictive humanised models. The ultimate objective is to engineer CAR‐T cells that function not just as targeted killers, but as microenvironmental engineers capable of dynamically and safely reshaping the tumour landscape to support sustained immune activity.

Navigating the translational valley of death for integrated CAR‐T therapies. The vision of ‘integrated engineering’ presents formidable technical, biological, and regulatory challenges that constitute a significant translational gap. First, manufacturing complexity escalates substantially with each additional genetic module incorporated into the cell product. This increases the risk of insertional mutagenesis, genotoxicity, and unpredictable interactions between engineered circuits, potentially affecting cell potency, persistence, or safety. The immunogenicity of synthetic components may trigger host immune responses that eliminate the engineered cells, as suggested by the limited persistence observed in some allogeneic trials [191]. Second, preclinical models often fail to predict human efficacy. Immunocompromised mouse xenografts lack a fully functional human immune system and cannot replicate the complex stromal and immunosuppressive networks of human TMEs. While humanised mouse models and 3D organoid systems are improving, their predictive value for the durability of response and the emergence of resistance mechanisms remains limited. Third, clinical trial design and patient selection become increasingly complex. For therapies targeting multiple antigens or TME factors, how does one define the appropriate patient population? What biomarkers predict response to a metabolically enhanced CAR‐T cell versus one armoured with cytokines? The lack of predictive biomarkers is a major bottleneck, as evidenced by the variable responses seen even with promising targets like CLDN18.2 [121, 123]. Finally, regulatory pathways for these ‘living drugs’ with multiple engineered functions are undefined. Defining critical quality attributes (CQAs) for a cell product with a logic gate, a metabolic switch, and a payload is exceptionally difficult. Agencies will require new frameworks to assess not only identity, purity, and potency, but also the dynamic interplay and stability of these functions in vivo. Overcoming these barriers will require close collaboration between basic scientists, clinical trialists, and regulators, as well as a commitment to iterative learning from early‐phase human studies, even those that do not meet traditional efficacy endpoints.

The vision of ‘integrated engineering,’ while promising, introduces formidable technical and regulatory hurdles. Combining multiple genetic modifications into a single cellular product substantially increases the complexity of manufacturing, quality control, and safety monitoring. The risk of insertional mutagenesis, genotoxicity, and unpredictable interactions between engineered modules grows with each added component. This is particularly pertinent for epigenetic reprogramming strategies, where stable genetic modifications intended to enforce a durable memory phenotype must be carefully balanced against the risks of altering fundamental transcriptional programs in unpredictable ways. Furthermore, the immunogenicity of synthetic receptors or bacterial‐derived enzymes may lead to host immune responses that eliminate the engineered cells, limiting their persistence. From a regulatory standpoint, defining the critical quality attributes (CQAs) for such multifaceted ‘living drugs’ is exceptionally difficult. The field must develop new frameworks to assess not only the product's identity, purity, and potency but also the dynamic interplay and stability of its engineered functions in vivo. Finally, the economic and accessibility barriers to producing these highly personalised, technologically advanced therapies cannot be overlooked.

The clinical application of engineered CAR‐T cells depends on precise toxicity management and real‐time monitoring. Clinical data indicate a trade‐off in which enhanced potency correlates with increased toxicity; in BCMA‐targeted CAR‐T therapy, greater persistence has been associated with higher neurotoxicity rates [174, 229, 230]. Emerging evidence suggests that neurotoxicity involves sustained microglial activation rather than solely cytokine surges, and transient microglial modulation restored cognitive function in preclinical models [169]. Durable responses in hematologic malignancies—including high response rates with bispecific BCMA/GPRC5D CAR‐T cells and salvage therapy efficacy after post‐transplant relapse—underscore the therapeutic capacity of CAR‐T technology [173, 176, 183]. Nonetheless, relapse driven by antigen escape and TME suppression remains frequent, reinforcing the need for multi‐targeting and resistance‐focused strategies. In solid tumours, signals from regional delivery approaches and new targets are encouraging, yet TME‐induced antigen modulation and deep immunosuppression persist, as shown by the limited activity of EGFRvIII CAR‐T cells with PD‐1 blockade in recurrent glioblastoma [106, 158, 180, 231, 232, 233]. Rational clinical application of co‐engineering principles—combining regional delivery, TME‐normalising agents, and armoured CAR‐T constructs—will be required to address these constraints.

Section 7 outlines how multiscale analytical and engineering platforms have moved CAR‐T development toward a rational design framework for solid tumours. Advanced imaging and spatial multi‐omics now provide mechanistic detail that guides construct and regimen optimisation, from tuning synapse biophysics to designing combinatorial strategies that disrupt immunosuppressive tissue architectures [195, 198, 209]. These datasets have been translated into engineering action: genome‐wide CRISPR screening identified edits such as SOCS1 and PRODH2 that enhance cellular fitness, while base and prime editing enabled epitope‐specific modifications crucial for safety and therapeutic window creation [67, 200, 202, 234, 235, 236]. These principles support next‐generation cellular architectures, including logic‐gated receptors and TME remodelling, payload‐armed CAR‐T cells, and are reshaping the pipeline through advances in allogeneic manufacturing, alternative cell sources, in vivo delivery platforms, and real‐time monitoring technologies [204, 206, 208, 214]. Systematic integration of insights across scales—from nanoscopic synapse imaging to tissue‐level profiling—is necessary to build adaptive cellular therapeutics capable of navigating and overcoming layered barriers in solid tumours.

Key research priorities are becoming clear. Dynamic, in vivo functional mapping should replace static biomarker discovery by integrating ultra‐sensitive liquid biopsy (HDSCA‐HemeCAR) and activatable granzyme B probes (G‐SNAT‐Cy5) with longitudinal multi‐omics to decode CAR‐T fate and the evolving TME [171, 172]. Receptor engineering should extend beyond dual‐antigen logic toward multi‐input sensory systems that conditionally activate based on TME signals such as hypoxia or protease activity. Universal targeting strategies that address heterogeneity, exemplified by the Myc‐pHLIP system for in situ tagging, warrant refinement for clinical use [159]. Manufacturing innovation remains essential, including rapid non‐viral platforms (e.g., ENI) and scaling of off‐the‐shelf allogeneic products to enable complex pre‐manufacturing edits [62, 64]. Predictive preclinical models such as 3D‐bioprinted vascularised tumour platforms should be further developed to support screening of complex combinations [162].

Looking forward, the field is poised to leverage AI and machine learning to accelerate the design of optimal CAR constructs and predict patient‐specific response patterns. The integration of real‐time biosensors for metabolites or TME factors into CAR‐T cells themselves could enable fully autonomous, self‐tuning cellular therapeutics. The convergence of cutting‐edge tools—advanced delivery systems like targeted LNPs and bacterial OMVs, real‐time monitoring via trackable reporters, computational design, and scalable manufacturing—with the core principles of integrated engineering outlined here will be critical to finally unlocking the full potential of CAR‐T therapy for the vast majority of cancer patients with solid tumours. The expansion into autoimmune diseases further demonstrates the platform's versatility and underscores the importance of safety‐engineered controls as these powerful cells are deployed against new classes of diseases.

## 9 Conclusion

This review synthesises the current trajectory of CAR‐T cell therapy, charting its evolution from a potent tool for hematologic malignancies toward a sophisticated, integrated platform aimed at overcoming the formidable barriers of solid tumours. The core contribution lies in framing the challenge not as a singular deficit but as a sequential series of obstacles—imprecise activation, metabolic fragility, antigenic escape, and a profoundly immunosuppressive TME—that demand a cohesive, multilayered engineering response. The evidence consolidates around a paradigm of ‘integrated intelligence,’ where advances in controllable receptor design, intrinsic metabolic‐epigenetic reprogramming, and active TME remodelling must be synergistically combined to create adaptive cellular therapeutics capable of co‐evolving with the dynamic tumour landscape.

The clinical and translational imperative is clear: to bridge the efficacy gap between hematologic and solid tumours, future efforts must translate these combinatorial engineering principles into viable clinical strategies. While clinical successes in multiple myeloma and other blood cancers validate the platform's foundational power, the limited efficacy in solid tumours underscores the necessity of the integrated approaches discussed, such as combining regional delivery with TME‐modulating agents and logic‐gated targeting. However, significant uncertainties remain, including the long‐term safety of complex genetic edits, the scalability of biomaterial and manufacturing innovations, and the predictive value of preclinical models for human TME complexity. Moving forward, priority should be given to the clinical validation of rational co‐engineering combinations, the development of dynamic in vivo monitoring to guide therapy, and the creation of scalable ‘off‐the‐shelf’ platforms to improve accessibility. By systematically integrating precision, resilience, and environmental remodelling, this roadmap provides a tangible pathway to extend the transformative potential of CAR‐T therapy toward the broader landscape of human cancers.

## Author Contributions

Original draft preparation, allocation: Chao Yan, Zhijie Zhao and Ping He. Revision and editing: Liming Wang, Shenglong Li and Tan Li. All authors have read and agreed to the published version of the manuscript.

## Funding

This work was supported by the Natural Science Foundation of Liaoning Province (2025JH2/101800397), China Health & Medical Development Foundation (chmdf2025‐JMWL‐07‐05) and Medical Science Research Fund of Beijing Medical and Health Foundation (YWJKJJHKYJJ‐ZH25042).

## Ethics Statement

The authors have nothing to report.

## Consent

The authors have nothing to report.

## Conflicts of Interest

The authors declare no conflicts of interest.

## Data Availability

The data that support the findings of this study are available from the corresponding author upon reasonable request.
